# Molecular insights into recognition of GUCY2C by T-cell engaging bispecific antibody anti-GUCY2CxCD3

**DOI:** 10.1038/s41598-023-40467-0

**Published:** 2023-08-17

**Authors:** Pragya Rampuria, Lidia Mosyak, Adam R. Root, Kristine Svenson, Michael J. Agostino, Edward R. LaVallie

**Affiliations:** 1 Biomedicine Design, Pfizer Inc., 610 Main St., Cambridge, MA 02139 USA; 2 Generate Biomedicines Inc, Cambridge, MA USA; 3 Pfizer Digital, Pfizer Inc., 1 Burtt Rd, Andover, MA USA

**Keywords:** Biological techniques, Drug discovery, Structural biology

## Abstract

The intestinal epithelial receptor Guanylyl Cyclase C (GUCY2C) is a tumor-associated cell surface antigen expressed across gastrointestinal malignancies that can serve as an efficacious target for colorectal cancer immunotherapy. Here, we describe a yeast surface-display approach combined with an orthogonal peptide-based mapping strategy to identify the GUCY2C binding epitope of a novel anti-GUCY2CxCD3 bispecific antibody (BsAb) that recently advanced into the clinic for the treatment of cancer. The target epitope was localized to the N-terminal helix H2 of human GUCY2C, which enabled the determination of the crystal structure of the minimal GUCY2C epitope in complex with the anti-GUCY2C antibody domain. To understand if this minimal epitope covers the entire antibody binding region and to investigate the impact of epitope position on the antibody’s activity, we further determined the structure of this interaction in the context of the full-length extracellular domain (ECD) of GUCY2C. We found that this epitope is positioned on the protruding membrane-distal helical region of GUCY2C and that its specific location on the surface of GUCY2C dictates the close spatial proximity of the two antigen arms in a diabody arrangement essential to the tumor killing activity of GUCY2CxCD3 BsAb.

## Introduction

Since their inception more than 30 years ago, therapeutic antibodies (Abs) have become the predominant class of new drugs developed in recent years^1,2^. During this time, antibody engineering methods to refine and optimize binding specificity and biophysical properties have become increasingly important^3^. These methods benefit greatly from detailed knowledge of the binding interface of the antibody with its target. The portion of the antigen involved in binding to the antibody is defined as the epitope of that antibody^4,5^. Detailed knowledge of the epitope can aid understanding of the mechanism of action of the antibody, the interactions that determine the binding strength of the antibody and how it can be modulated through site-specific modifications^6,7^. Mapping the epitope can help to differentiate between antibodies of varying efficacy. Information on the epitope is ever more essential to secure intellectual property rights for the development of antibody products, whether therapeutic or diagnostic^6^. Several methods have been devised for epitope mapping including the use of truncated proteins, synthesized peptides, X-ray crystallography^8^, nuclear magnetic resonance (NMR) spectroscopy^9,10^, mass spectrometry^11^ and electron microscopy analyses of antibody–antigen complexes. Some recent methods include bioinformatic analyses of large datasets derived from mutational scanning, protein display, and high-throughput screening experiments^3,12–16^.

Here, we have combined yeast display technology with in-silico epitope prediction tools, comparative structural modelling, and subsequent X-ray crystallography to unravel the binding epitope of anti-GUCY2C arm of the bispecific therapeutic antibody PF-07062119^17,18^. Yeast display is a powerful tool to express antigens and their variants and has proven very useful in expressing libraries with ~ 10^8^ diversity^19,20^. The eukaryotic yeast secretory system allows for expression of different forms of antigen on the surface in their native conformation^21^. Unlike stable mammalian cell expression, transforming and inducing expression of proteins on *Saccharomyces cerevisiae* is fast and efficient. Domain and epitope mapping using yeast surface display is also advantageous since it allows for measuring epitope interaction and antigen expression using flow cytometry^22,23^.

GUCY2C is a receptor expressed normally on the luminal surfaces of the intestinal epithelium and certain types of hypothalamic neurons^24,25^. The main function of GUCY2C is maintenance of intestinal homeostasis^24^. The role of GUCY2C in the brain is not well-defined but has been linked to appetite regulation and energy balance in the hypothalamus^24^. GUCY2C is activated by the hormones guanylin and uroguanylin and the heat stable (ST) enterotoxin from *Escherichia coli* to produce cyclic GMP (cGMP)^26–30^.

GUCY2C is a surface membrane glycoprotein consisting of an amino-terminal ECD, a single transmembrane helix, and a cytoplasmic region encompassing a kinase homology domain (KHD), a linker region, the catalytic domain, and a carboxy-terminal domain^26^. Among the members of the family of membrane-bound guanylyl cyclases only the crystal structure of the atrial natriuretic peptide receptor [ANP receptor, guanylyl cyclase-A (GC-A)] has been successfully generated^31^;* pdb* = *3A3K*^32^;* pdb* = *1dp4*^33^;* pdb* = *1T34.* Detailed structural information of GUCY2C has thus far remained elusive. While the intracellular domains of these family members have a sequence identity of ~ 55%, their ECD sequences are significantly less conserved, with sequence identity between the ECD of GUCY2C and GC-A only 19%. Another related protein for which the structure is available is that of the natriuretic peptide clearance receptor C (NPR-C)^34^;* pdb* = *1JDN & 1JDP*^35^;* pdb* = *1YK0.* While this receptor is not a guanylyl cyclase, the GUCY2C ECD has 34% amino acid sequence identity to the extracellular domain of the ANP receptor.

GUCY2C is widely expressed on colorectal cancer cells and other gastrointestinal tumors^24,25,36^. GUCY2C is ordinarily expressed strictly on the apical side of the intestinal tight junctions, whereas in colorectal cancer (CRC) and other gastrointestinal tumors it is expressed ectopically in other sites such as gastric, esophageal, and pancreatic tissues, making GUCY2C an appealing antigen for a biotherapeutic approach^17,25,37–39^. PF-07062119 is a bispecific monoclonal antibody with an anti-GUCY2C arm paired with an anti-CD3 arm. It was developed as a biotherapeutic for CRC and has shown efficacy in multiple CRC models^17^. The anti-GUCY2C arm of PF-07062119 was originally derived from mouse hybridomas^18^. The antibody was humanized and further optimized using structure-guided rational design coupled with phage display methods to improve stability, reduce polyreactivity and self-association potential, and reduce immunogenicity risk to make it suitable for clinical development^17,18^.

In this paper, we report on the mapping and identification of the binding epitope of the anti-GUCY2C arm of PF-07062119 bispecific antibody using chimeras of GUYC2C protein, displayed on the surface of yeast, with the result further corroborated by peptide-based ELISA. To gain molecular insights into antibody recognition that led to the development of this effective anti-GUCY2CxCD3 therapeutic, we structurally delineated the binding site of anti-GUCY2C arm on GUCY2C by X-ray crystallography. Our structural studies provided understanding of the details and the basic molecular principles of GUCY2C recognition that govern the full therapeutic potential of the T-cell engaging GUCY2CxCD3 antibody in cancer.

## Results

### Anti-GUCY2C bispecific antibody PF-07062119 binding to different species of GUCY2C

**Figure 1:**
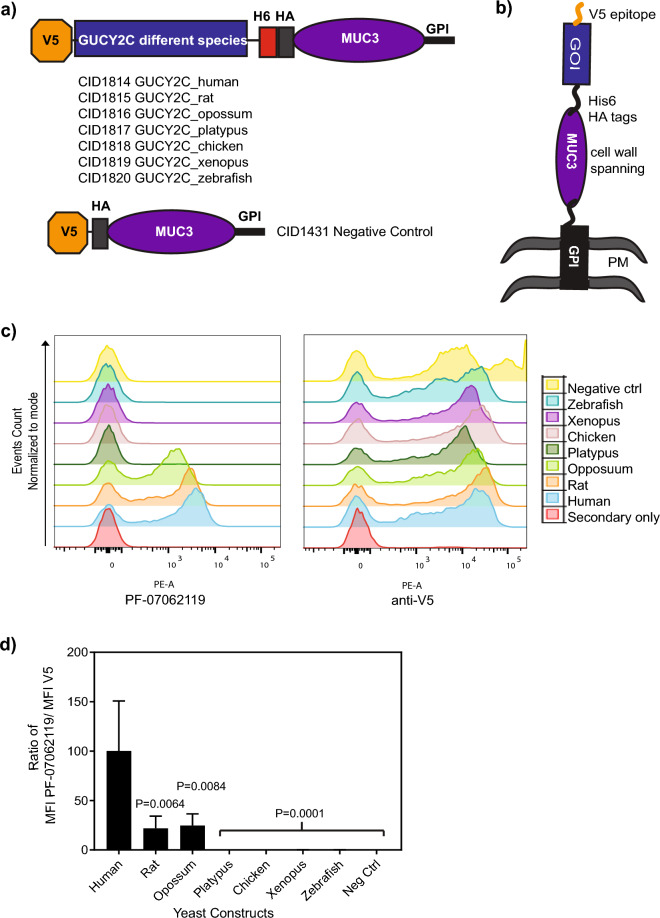
Anti-GUCY2c arm of PF-07062119 binds to human, rat and opossum GUCY2C expressing yeast cells. (**a**) Graphical representation of the GUCY2C expression constructs for human (CID1814), rat (CID1815), opossum (CID1816), platypus (CID1817), chicken (CID1818), frog (CID1819) and zebrafish (CID1820) and negative control (CID1431). (**b**) Depiction of the display orientation of the GUCY2C proteins on the surface of yeast cells, with the GPI anchor in the plasma membrane (PM), the Muc3 domain spanning the cell wall of yeast cells and the hexa-Histidine (H6), HA and N-terminal V5 tags exposed on the surface along with the gene of interest (different orthologues of GUCY2C protein). (**c**) *S. cerevisiae* cells were transformed with the constructs depicted in (a) and induced to express the different GUCY2C orthologs from the species listed above. Binding of PF-07062119 (left panel), and anti-V5 Ab (right panel) to different orthologues of GUCY2C was evaluated using flow cytometry. Histogram overlays of binding of antibodies to the different constructs are shown. Each GUCY2C orthologue is represented by one color on the histogram overlay indicated in the legend. (**d**) Ratio of median fluorescent intensity (MFI) of PF-07062119 to the MFI of anti-V5 Ab. Bars represent the mean ± standard deviation of 2 independent experiments, with triplicate samples in each experiment. Statistical significance is indicated by P values shown in the graph.

A strategy was devised to identify epitope regions for the anti-GUCY2C antibody PF-07062119 on human GUCY2C by substituting regions of GUCY2C sequence from a species homolog that does not bind the antibody. To guide the generation of relevant GUCY2C substitution mutations, it was necessary to screen the binding of PF-07062119 to GUCY2C proteins from a variety of species. Thus, cDNAs encoding GUCY2C ECD sequences from different species including human (CID1814), rat (CID1815), opossum (CID1816), platypus (CID1817), chicken (CID1818), frog (CID1819) and zebrafish (CID1820), were displayed on the surface of *S. cerevisiae* cells (Fig. 1a). Each GUCY2C ECD cDNA was fused at the N terminus to a sequence encoding the V5-epitope tag and at the C-terminus to sequences encoding His6 and HA tags (Fig. 1a,b). The displayed protein was designed with additional C-terminal sequences encoding muc3 protein followed by a glycosylphosphatidylinositol (GPI)-anchor sequence. Thus, the secreted protein was tethered to the yeast cell membrane via the GPI anchor and extended through the cell wall to the exposed cell surface by the extended structure of the muc3 segment (Fig. 1b). A control plasmid (CID1431) that was deleted for GUCY2C cDNA and encoded only the epitope tags fused to the muc3-GPI was used as a negative control for binding to anti-GUCY2C antibodies (Fig. 1a). The display level of the different species orthologue ECDs on the yeast surface and the binding of PF-07062119 to the displayed ECDs was measured using flow cytometry (Fig. 1c). On normalizing binding to display levels, it was found that PF-07062119 bound to human GUCY2C as expected and bound weakly to rat and opossum GUCY2C ECD but did not bind platypus, chicken, frog and zebrafish (Fig. 1d).

### Generation of human-chicken GUCY2C Chimeras and Patch mutants

To identify the residues to mutate in chimeras and patch mutants, a multiple sequence alignment was performed comparing human GUCY2C sequence to the GUCY2C orthologue sequences. The positions of amino acid residues that are identical between human, rat and opossum (the binders) and dissimilar in sequence of chicken, frog and zebrafish (non-binders) were identified (Supplementary Fig. S1a). These positions were examined on the GUCY2C structural models (Supplementary Fig. S2) to determine residues that were predicted to be surface-exposed and accessible for antibody binding (Supplementary Table S1), as well as residues that were predicted to be in close proximity to each other. These positions were chosen to be mutated in human GUCY2C to the amino acids of a non-binding species. Chicken GUCY2C sequence was chosen for the substitutions to generate chimeras and patch mutants because it was the species that showed no binding to PF-07062119 and was closest to human GUCY2C by sequence identity. Platypus sequence was excluded from the multiple sequence alignment because of the 100 extra amino acids that were present in the platypus GUCY2C sequence but were not present in any of the other GUCY2C sequences of the species under study. Results from three different antigen prediction algorithms-Bepipred Linear Epitope Prediction, Emini Surface Accessibility Prediction and Kolaskar & Tongaonkar Antigenicity, were used to guide the selection of the regions to be targeted with amino acid substitutions. The regions that scored highest and were detected by all three tools were chosen to be modified from human to chicken GUCY2C residues in the chimeras (Supplementary Fig. S1b). A total of 6 patch mutants (CID1868–1873) and 8 chimeras (CID1874–1881) were generated.

### Anti-GUCY2C bispecific antibody PF-07062119 binds to residues 73–87 of human GUCY2C

**Figure 2:**
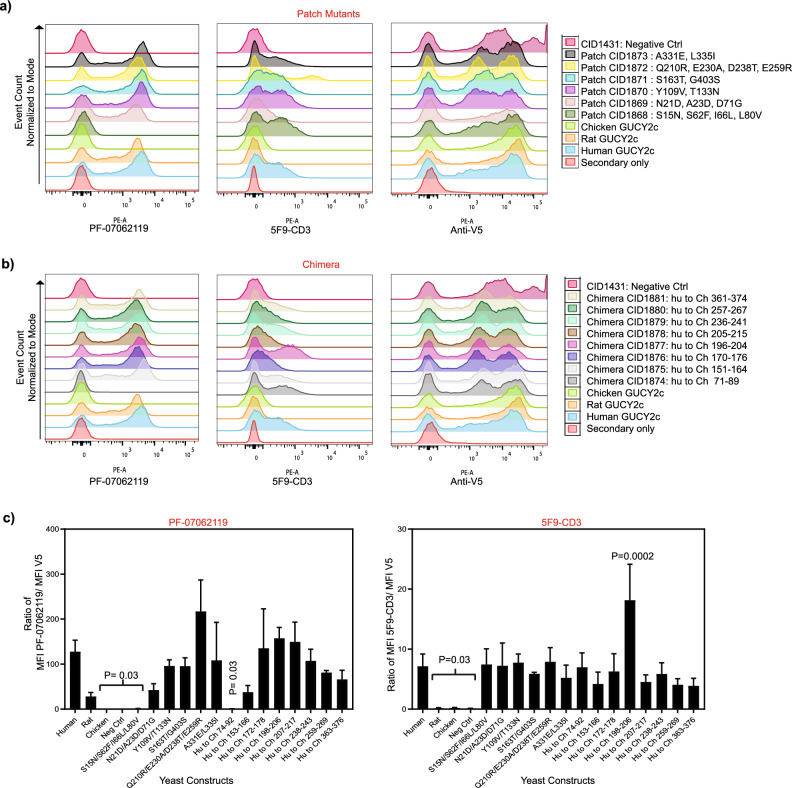
PF-07062119 binds to residues 74–92 of GUCY2C. (**a**) Flow cytometry analysis of yeast cells expressing human/chicken GUCY2C patch mutants (Patch CID1868: S15N, S62F, I66L, L80V; Patch CID1869: N21D, A23D, D71G ; Patch CID1870: Y109V, T133N; Patch CID1871: S163T, G403S; Patch CID1872: Q210R, E230A, D238T, E259R; Patch CID1873: A331E, L335I) on the cell-surface along with human (CID1814), rat (CID1815) and chicken (CID1818) wild-type GUCY2C and CID1431 expressing yeast cells as positive and negative controls. Patch mutants contain 2–4 individual amino acid substitutions of the human GUCY2C sequence with the cognate amino acids found in the chicken GUCY2C sequence and predicted to be surface-exposed and clustered together in the structural model.. Histogram overlays of binding of antibodies PF-07062119 (left panel), 5F9-CD3 (center left panel) and anti-V5 Ab (right panel) to the different constructs are shown. Each patch mutant is represented by one color on the histogram overlay as indicated in the legend. (**b**) Flow cytometry analysis of yeast cells expressing same control yeasts as 2a) and human/chicken GUCY2C chimeras in the regions indicated (CID1874: substitute 74–92 in human with chicken residues 71–89; CID1875: substitute 153–166 in human with chicken residues 151–164; CID1876: substitute 172–178 in human with chicken residues 170–176; CID1877: substitute 198–206 in human with chicken residues 196–204; CID1878: substitute 207–217 in human with chicken residues 205–215; CID1879: substitute 238–243 in human with chicken residues 236–241; CID1880: substitute 259–269 in human with chicken residues 257–267; CID1881: substitute 363–376 in human with chicken residues 361–374. Chimeras contain varying lengths of amino acid sequence from chicken GUCY2C substituted for the human sequence. Histogram overlays of binding of antibodies PF-07062119 (left panel), 5F9-CD3 (center panel) and anti-V5 Ab (right panel) to the different constructs are shown. Each chimera is represented by one color on the histogram overlay as indicated in the legend. (**c)** Ratio of MFI of PF-07062119 to the MFI of anti-V5 Ab (left) and ratio of MFI of 5F9-CD3 to the MFI of anti-V5 Ab (right). Bars represent the mean ± standard deviation of 2 independent experiments, with triplicate samples in each experiment. Statistical significance is indicated by P values shown in the graph.

Binding of PF-07062119 and 5F9-CD3 (an anti-GUCY2C antibody that binds human GUCY2C at an epitope distinct from PF-07062119^40^) to the chimeras and patch mutants along with controls were measured by flow cytometry. These controls were human GUCY2C (CID1814), rat (CID1815) and chicken GUCY2C (CID1818) and negative control yeast (CID1431). Display level of the different constructs on the surface of the yeast was measured by anti-V5 tag antibody binding (Fig. 2a,b). Binding of PF-07062119 to CID1868 and CID1874 was significantly reduced compared to binding to native human GUCY2C (Fig. 2a–c). These data suggested that residues mutated in patch mutant CID1868-S15N, S62F, I66L, L80V; (mature protein numbering here and throughout the text) and chimera CID1874 (residues 74–92) were likely to be part of the epitope of PF-07062119. 5F9-CD3 did not show the same reduced binding to CID1868 and CID1874 (Fig. 2a–c) indicating that this loss in binding of PF-07062119 to these two variants was not due to structural disruptions. Both PF-07062119 and 5F9-CD3 did not bind to chicken GUCY2C and negative control CID1431 as was previously observed. Also, of note is that L80V is a mutation that is common between CID1868 and CID1874.

Peptide mapping was performed to determine the epitope of PF-07062119 on the GUCY2C extracellular domain. For this approach, 20-mer peptides, with 15 amino acid overlaps were generated, covering the entire ECD into the transmembrane domain of GUCY2C consisting of amino acids 1–440 (GenPept Accession No. NP-004954). These peptides were arrayed on a plate and assessed for binding to PF-17062119 by DELFIA. PF-07062119 showed strong binding to GUCY2C ECD peptides 19 and 20 (sequences NSGDCRSSTCEGLDLLRKIS and RSSTCEGLDLLRKISNAQRM) as measured by time resolved fluorescence (TRF) while TRF binding signal against all other peptides was at or near background levels (Supplementary Table S2). The sequence, NSGDCRSSTCEGLDLLRKISNAQRM is spanned by these two overlapping peptides. The region of overlap among the peptides is RSSTCEGLDLLRKIS indicating that the specific binding epitope is within this region.

**Figure 3:**
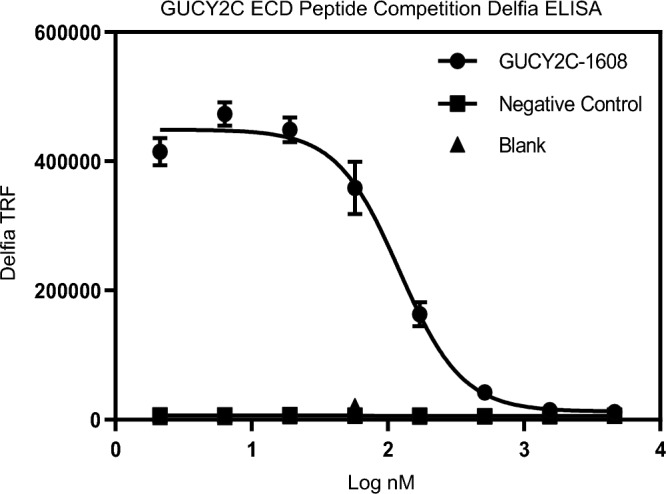
Peptide 19 contains the specific epitope where PF-07062119 binds on the full GUCY2C ECD. Competition ELISA was performed with plates coated with GUCY2C-ECD (1ug/ml), incubated with EC80 of GUCY2C-1608 or negative control Ab with serial dilutions of Peptide 19 starting at 10 ug/ml, diluted 1:3. Graph shows time resolved fluorescence (TRF) of GUGY2C-1608 versus negative control and blank on y-axis and concentration of Peptide 19 on x-axis. Bars represent the mean ± standard deviation of duplicate samples. The experiment was run twice independently.

GUCY2C peptide 19 was shown to effectively compete for PF-17062119 binding against the full ECD in a competition assay (Fig. 3), suggesting that peptide 19 contains the specific epitope where PF-07062119 binds on the full GUCY2C ECD.

**Figure 4:**
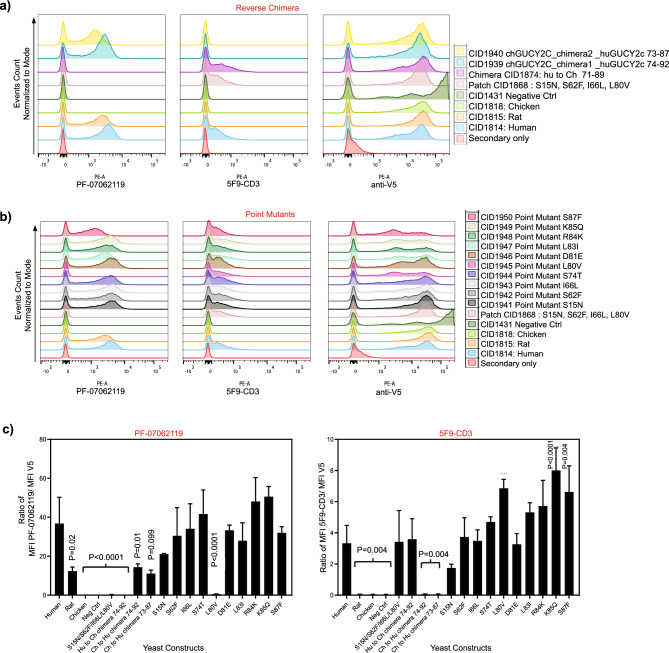
Reverse chimeras confirm residues 73–87 of hu-GUCY2C constitute the epitope of the anti-GUCY2C arm of PF-07062119. (**a**) Flow cytometry analysis of yeast cells expressing reverse chimeras (CID1939 chGUCY2C_chimera1 _huGUCY2C 74–92; CID1940 chGUCY2C_chimera2 _huGUCY2C 73–87, where human GUCY2C amino acid residues 74–92 and 73–87 are substituted into chicken GUCY2C sequence) on the cell-surface. Human (CID1814), rat (CID1815) and chicken (CID1818) GUCY2C, patch mutant CID1868, chimera CID1874 and CID1431 expressing yeast cells were used as controls. Histogram overlays of binding of antibodies PF-07062119 (left panel), 5F9-CD3 (center panel) and anti-V5 Ab (right panel) to the different constructs are shown. Each yeast construct is represented by one color on the histogram overlay indicated in the legend. (**b**) Flow cytometry analysis of yeast cells expressing the point mutants (CID1941 huGUCY2C_S15N; CID1942 huGUCY2C_S62F; D1943 huGUCY2C_I66L; CID1944 huGUCY2C_S74T; CID1945 huGUCY2C_L80V; CID1946 huGUCY2C_D81E; CID1947 huGUCY2C_L83I; CID1948 huGUCY2C_R84K; CID1949 huGUCY2C_K85Q; CID1950 huGUCY2C_S87F, where single amino acids from human GUCY2c are substituted with its chicken GUCY2c counterpart). Human (CID1814), rat (CID1815) and chicken (CID1818) GUCY2C, patch mutant CID1868, and CID1431 expressing yeast cells were used as controls. Histogram overlays of binding of antibodies PF-07062119 (left panel), 5F9-CD3 (center panel) and anti-V5 Ab (right panel) to the different constructs are shown. Each yeast construct is represented by one color on the histogram overlay indicated in the legend. (**c**) Ratio of MFI of PF-07062119 to the MFI of anti-V5 Ab (left) and ratio of MFI of 5F9-CD3 to the MFI of anti-V5 Ab (right). Bars represent the mean ± standard deviation of 2 independent experiments, with triplicate samples in each experiment. Statistical significance is indicated by P values shown in the graph.

To further confirm the results from the yeast display and peptide ELISA analysis, reverse chimeras and point mutants were generated in an attempt to confer binding of PF-07062119 to chicken GUCY2C by substituting with human GUCY2C amino acids at the positions implicated (Fig. 4). The reverse chimeras comprised of chicken GUCY2C residues 71–89 and 70–84 replaced by human 74–92 (CID1939) and 73–87 (CID1940), respectively, showed restoration of binding to PF-07062119 compared to wild-type chicken GUCY2C which showed no binding to PF-07062119 (Fig. 4a,c). The reverse chimeras did not bind to 5F9-CD3, like the wildtype chicken GUCY2C (Fig. 4a,c). Of the ten point-mutants tested, only one mutant L80V (CID1945) showed significantly reduced binding to PF-07062119 when compared to binding of PF-07062119 to huGUCY2C (Fig. 4b,c), indicating that L80 is one of the pivotal residues in the binding epitope of PF-07062119. Thus, the combination of yeast display and peptide ELISA confirmed that residues 73–87 are part of the epitope of PF-07062119.

### Crystal structure of a GUCY2C-peptide in complex with anti-GUCY2C-scFv

**Figure 5:**
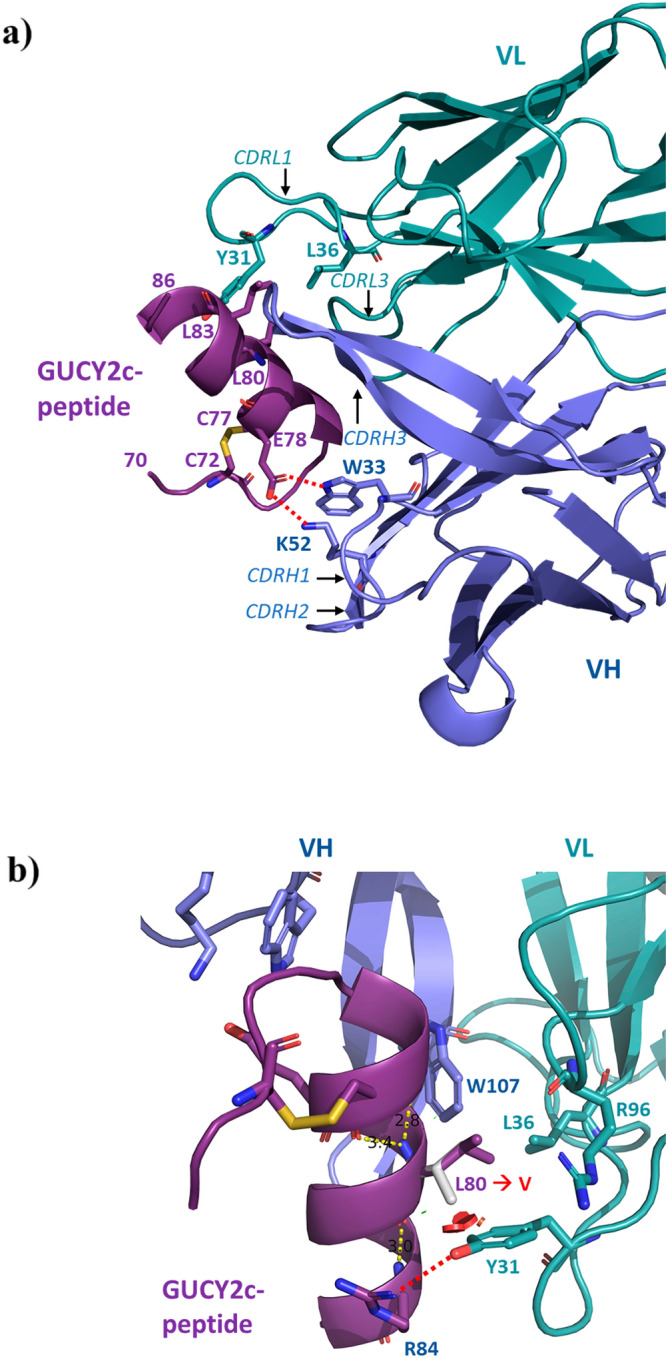
Binding interface between ^68^GUCY2C-peptide^87^-and aGUCY2C-scFv. (**a**) Ribbon diagram showing the CDR-loops from VH and VL (blue and cyan) in direct contact with the GUCY2C-peptide (magenta). Disulfide bridge C72-C77 in GUCY2C and the key interacting residues at the interface are shown as sticks. Polar interactions shown by dashed lines. (**b**) Close-up view of the binding interface showing the location of Leu 80 to Val mutation studied in this article. Leu 80 is shown as magenta sticks and modeled Val 80 as white sticks. Solid red spheres indicate the predicted clash between the side chain rotamer of Val 80 and the aromatic ring of Tyr 31 from CDR-L1. Hydrogen bonding between Tyr 31 and Arg 84 of GUCY2C is shown by red dashed lines.

Guided by the peptide mapping and yeast display results which indicated that PF-07062119 is directed against the GUCY2C epitope contained in the amino acid region 68–87, we co-crystallized and determined the structure of anti-GUCY2C-scFv (aGUCY2C-scFv) bound to a chemically synthesized peptide covering this region ^68^NSGDCRSSTCEGLDLLRKIS^87^. The crystals contained two copies of complexes in the asymmetric unit displaying very similar conformations and structures with root mean square deviation (r.m.s.d.) of 0.49 Å for 120 Ca-pairs (Supplementary Fig. S3a). Therefore, for the purpose of structure description we will refer to one representative copy of this complex in the crystal. The GUCY2C-peptide ^68^NSGDCRSSTCEGLDLLRKIS^87^ (structurally ordered residues are underlined) adopts mostly an α-helical confirmation which is constrained by the disulfide bond ^72^Cys–Cys^77^ to the N-terminal portion of the peptide (Fig. 5a). The peptide binds to a CDR cleft formed by the heavy and light chains of the variable fragment extending from one side, CDR-H2, to the other, CDR-L1. aGUCY2C-scFv utilizes all CDR loops to contact the GUCY2C-peptide except CDR-L2. A mosaic of contacts at the binding interface includes hydrophobic, polar, and charged residues making complementary interactions. The total binding surface area buried under interaction is ~ 1235 Å^2^, which is fairly extensive and within the range of typical antibody-antigen interactions. Amino acids of the GUCY2C-peptide and aGUCY2C-scFv that are in contact with each other within 3.8 Å are defined as the binding epitope and paratope residues respectively and are listed in Supplementary Table S3. The aGUCY2C-scFv epitope residues contributing over 75% of their surface areas to the binding interface are listed in Supplementary Table S4.

The observed molecular contacts at the binding interface are in accord with the principal results from our GUCY2C chimera mutagenesis studies. Among the residue positions in the GUCY2C-binding site that were probed in these studies Leu 80 was the only one that was largely intolerant to valine substitution (L80V, Fig. 4). Leu 80 is buried in the hydrophobic pocket (< 22% solvent accessibility, Supplementary Table S4) forming prominent contacts with aGUCY2C-scFv through Trp 107 from CDRH3, Tyr 31 and Leu 36 from CDRL1 and Arg 96 from CDRL3 (Fig. 5b, Supplementary Table S3). Substitution of the leucine to the shorter valine side chain at this position will likely weaken or eliminate these packing interactions. Of particular note is a predicted steric clash between the side chain rotamer of Val 80 and the aromatic ring of Tyr 31 that makes hydrogen bonding interaction with Arg 84 of GUCY2C (Fig. 5b). This clash may facilitate torsion of Tyr 31 away from GUCY2C resulting in loss of its hydrogen bonding with Arg 84 and further destabilization of the binding site.

The second mutated residue that is part of the GUCY2C binding epitope is Leu 83. Substitution of Leu 83 to isoleucine led to the mutant protein L83I with slightly reduced but largely retained binding activity (Fig. 4). Leu 83, located directly above Leu 80 (Fig. 5a) but more deeply buried in the pocket (< 12% solvent accessibility, Supplementary Table S4), makes packing interactions with E104 and G105 from CDRH3 and L36 from CDRL1. Since both Leu and Ile have similar sizes and polarities the overall interactions in this binding pocket will be largely preserved explaining a diminutive effect on the binding activity of the L83I mutant.

### Crystal structure of GUCY2C-ECD in complex with aGUCY2C-scFv

To gain further molecular insight into the binding mode of aGUCY2C-scFv and to understand if structural details of the aGUCY2C-scFv epitope-paratope binding derived from the isolated GUCY2C-peptide are recapitulated in the context of the complete ECD protein we co-crystallized the full length GUCY2C-ECD with aGUCY2C-scFv and attempted structure determination of this complex. However, progress in our structure determination was hampered by two factors: (1) limitation of crystal diffraction to ~ 3.5 Å resolution and (2) difficulties in building an adequate template of GUCY2C model for molecular replacement search. Although the templates built from the low homology structures of ANPR and NPR-C yielded molecular replacement solutions, the two above factors combined prevented us from obtaining electron density maps of sufficient quality for a reliable model of the complete GUCY2C-ECD. The subsequent appearance of the AlphaFold (AF) protein structure prediction platform^41^ was game-changing and eventually allowed us to solve the phase problem, obtain high quality electron density maps and build an atomic model of the complete GUCY2C-ECD-aGUCY2C-scFv structure at this resolution. Pairwise superpositions of the final refined structure of GUCY2C-ECD with the AF-model and ANPR-ECD and NPR-C-ECD structures (Supplementary Fig. S4) clearly demonstrated that the best fit to the GUCY2C structure was provided by the AF-model (r.m.s.d. value of 0.91 Å for 334 aligned Ca atoms; Supplementary Fig. S4a) compared to ANPR and NPR-C (r.m.s.d. values of 2.78 Å for 291 Cα pairs and of 2.72 Å for 294 Cα pairs, respectively; Supplementary Fig. S4b,c), explaining why the structures of ANPR or NPR-C were not of sufficient homology to allow molecular modeling at this resolution.

**Figure 6:**
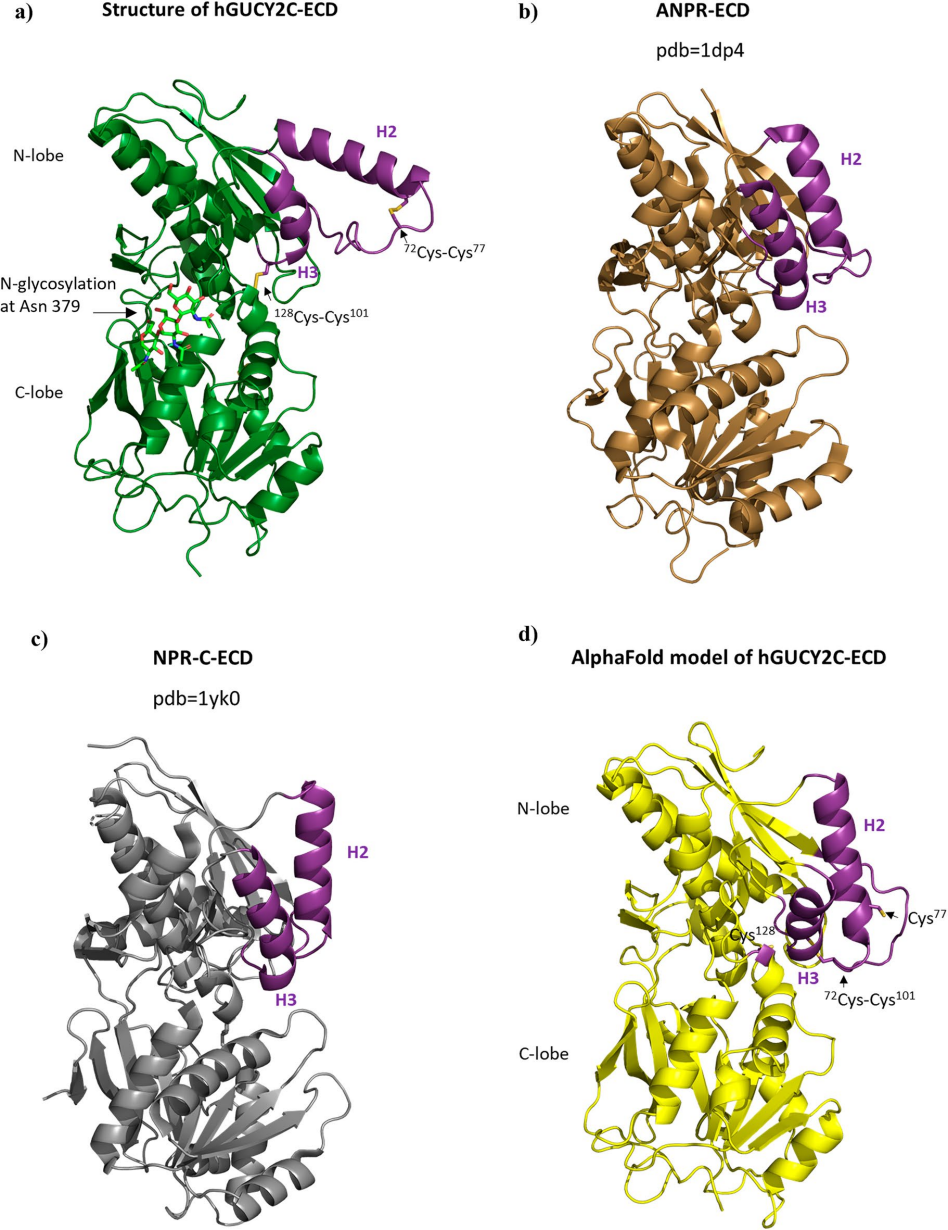
Ribbon diagram comparison of GUCY2C-ECD structure with the AF model and the structures of low homology member-associated ANPR-ECD and NPR-C-ECD. (**a**) Structure of GUCY2C-ECD is shown in green. Disulfide bonds around helices H2 and H3 are shown as sticks and labeled. Green sticks represent sugar units attached at the N-glycosylation site Asn 379. (**b**) Structure of ANPR-ECD is shown in brown. (**c**) Structure of NPR-C-ECD is shown in grey. (**d**) AF model of GUCY2C-ECD is shown in yellow. The assignment of disulfide paring around helices H2 and H3 (one disulfide and two free cysteines shown as sticks and labeled) is inaccurate and different from that seen in the crystal structure. Structural differences in positions of helices H2 and H3 are highlighted in magenta in (**a**–**d**).

**Figure 7:**
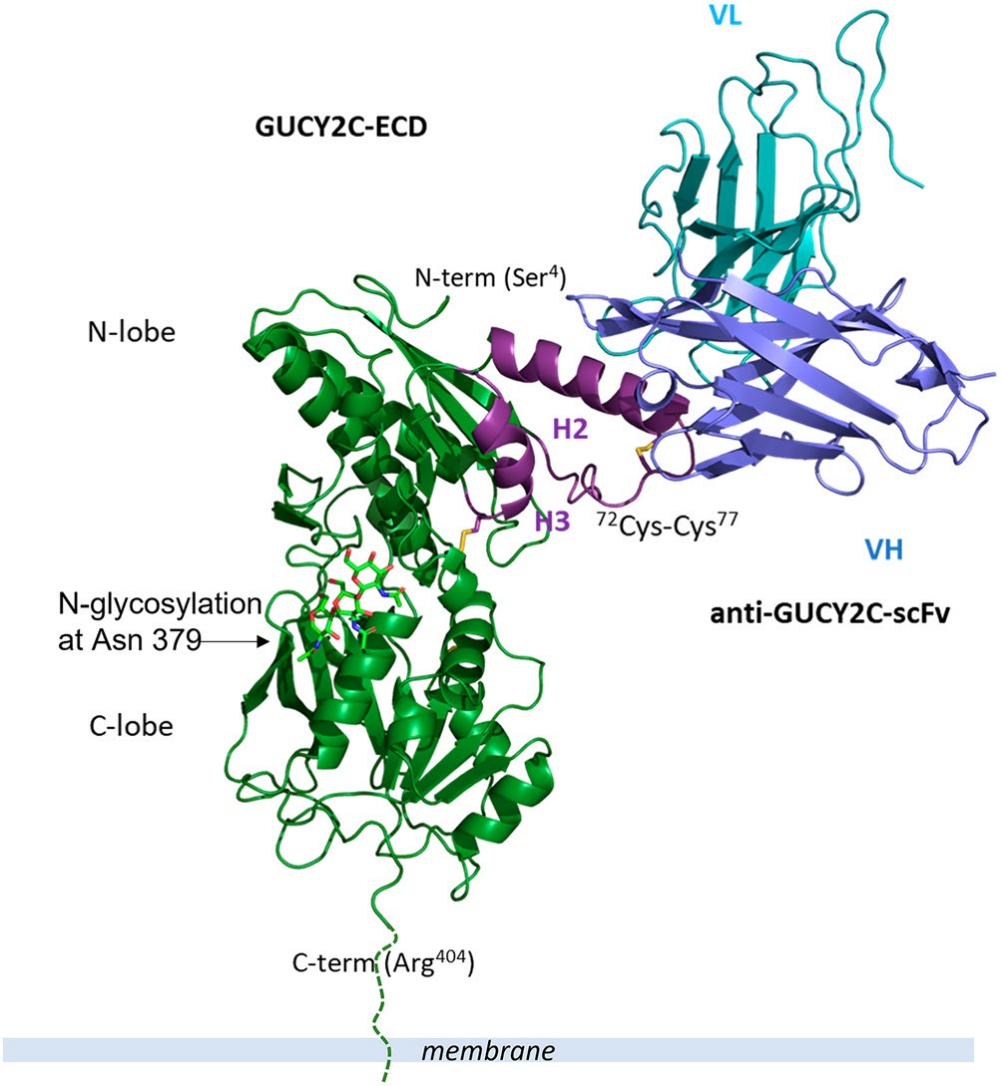
Overall view of the interaction between GUCY2C-ECD and anti-GUCY2C-scFv. GUCY2C is colored in green and magenta and anti-GUCY2C-scFv in blue and cyan. The terminal residues of GUCY2C-ECD that are seen in the electron density map at the N- and C-termini are labeled. N-glycosylation site at Asn 379 is indicated. The three sugar units for which the electron density is present are shown in green sticks. The extrapolated C-terminus of GUCY2C is modelled; attachment to the membrane is shown as dashed line.

As expected from the predicted AF model used in molecular replacement, the GUCY2C-ECD possesses a bi-lobed fold related to that found in the low sequence homology member-associated ANPR and NPR-C, with N- and C-lobe each covering a central β-sheet flanked by α-helices (Fig. 6). However, comparison of the GUCY2C-ECD structure (Fig. 6a) with the above two reference structures (Fig. 6b,c), as well as with the AF model (Fig. 6d), clearly showed that the GUCY2C helix H2 encompassing ^68^GUCY2C-peptide^87^ adopts a different orientation and a more distal position with respect to the rest of the domains. The structure of GUCY2C-ECD bound to aGUCY2C-scFv (Fig. 7) revealed that such spatial arrangement of helix H2 allows the binding epitope residues of GUCY2C to be presented on the outside face of the helix enabling the recognition by aGUCY2C-scFv with two prominent features. First, aGUCY2C-scFv binds exclusively to helix H2 avoiding interaction with the other parts of the ECD structure. Second, the binding epitope resides on the most distal part of the structure relative to the membrane (Fig. 7), underlying the importance of its location for effective GUCY2CxCD3 immunotherapeutic target as discussed in more detail in “Discussion” section.

Further examination of the binding mode of aGUCY2C-scFv to GUCY2C-ECD (Fig. 7) revealed that in the context of the full length GUCY2C-ECD, helix H2 retained the same binding site characteristics as the isolated ^68^GUCY2C-peptide^87^ bound to aGUCY2C-scFv showing remarkable similarity to the binding interface of the latter (Supplementary Fig. 3b). Based on the above observations we surmised that the ^68^GUCY2C-peptide^87^ region defines the entire aGUCY2C-scFv binding epitope, in accord with the yeast display results and the fairly large size of the binding interface calculated from the high resolution ^68^GUCY2C-peptide^87^-aGUCY2C-scFv structure.

Importantly, in the absence of the apo structure of GUCY2C, the question if the structural changes observed around the binding epitope represent the apo conformation of GUCY2C or resulted from antibody binding cannot be fully addressed. However, detailed analysis of the GUCY2C disulfide pattern around helices H2 and H3: Cys72–Cys77 and Cys101–Cys128 argues against the second, latter scenario. The above connectivity pattern of disulfide bridges is very divergent from that observed in the structures of ANPR and NPR-C (Supplementary Fig. S5). In the latter two, helix H2 is anchored to helix H3 by the conserved disulfide bridge (Supplementary Fig. 5b,c) stabilizing the close positioning of helix H2 to helix H3 and ensuring its structural proximity to the rest of the ECD. In the case of GUCY2C, four cysteine residues Cys72, Cys77 and Cys101, Cys128 surrounding helices H2 and H3 (Supplementary Fig. S5a) occupy positions in the sequence and structure that cannot be accommodated by the conserved disulfide linkage in ANPR and NPR-C. Instead, Helix 3 is anchored to the β-sheet of the N-lobe by Cys101–Cys128, and Helix 2 is disulfide constrained by Cys-72–Cys77 but lacks disulfide linkage to Helix 3 projecting away and out from the core of the structure. This observation suggests that the novel cysteine paring observed in GUCY2C could be a key determinant for ensuring the helix H2 specific location in the structure. Moreover, the fact that both disulfides are not in a location likely to be affected by binding of aGUCY2C-scFv argues against induced conformational changes suggesting instead that it is an inherent feature of the apo conformation of GUCY2C preconfigured for binding. In this regard, it is not uncommon that the divergent (non-conserved) disulfide sites often associate with variable structural features that are recruited for differentiation or specialization of protein function, therefore supporting the notion that there could be significant local changes in the apo structure of GUCY2C due to the presence of more disulfide bonds and new disulfide patterns compared to its homologues ANPR and NPR-C.

## Discussion

GUCY2C, a surface membrane glycoprotein expressed on gastrointestinal tumor cells, represents an attractive target for T-cell immunotherapy against cancer. This approach requires creation of a highly specific monoclonal antibody and a detailed understanding of the epitope of the antibody to assist in engineering and optimization of potent cytotoxic bispecific therapeutic mAbs. In this study we describe the mapping, identification, and structural delineation of the epitope of the anti-GUCY2C arm of the bispecific antibody PF-2610019. This effort was hindered by the fact that GUCY2C falls into the category of difficult-to-crystallize antigens. There are no previous structures available of GUCY2C or any closely related family homologs. The closest family homolog with an identified structure is atrial natriuretic peptide receptors (ANPR)^32^. The low sequence identity (17%) between ANPR and GUCY2C ECD’s^42^ dissuaded us from using ANPR sequences to create chimera and point mutants of GUCY2C. The finding that PF-2610019 bound selectively to only certain species of GUCY2C allowed for the rational design of chimeras and patch mutants with confidence that the native folding would be preserved, along with guidance from the GUCY2C structural model and in silico epitope prediction tools.

The higher sequence identity among GUCY2C species orthologues helped to reduce the number of chimeras needed to identify the epitope. In addition, a combination of large-scale statistical analysis of antigen–antibody cocrystals from the protein databases and in-silico analysis has helped to define physicochemical, structural, and geometrical aspects of epitopes that allowed further narrowing of the likely regions of antibody binding. One commonly defined feature of an epitope is its surface accessibility. Patch mutants were chosen based on this feature and structural proximity observed on the structural model of GUCY2C that was generated.

Mutational Scanning has been used to map a variety of protein–protein interactions but it is a low throughput method and is able to map only the functional epitope rather than the entire epitope as the effect of a single mutation on binding may not be detected even when the residue is within the epitope. Also, inferring the results of mutational scanning can be complicated because it can be difficult to decipher whether a mutation is disruptive because it is part of the epitope or because it destabilizes the overall fold of the protein. Van Blarcom et al.^23^ used a combination of a rationally designed antigen library, quantitative selection through yeast surface display and in-depth computational analysis of the enriched populations through high-throughput next generation DNA sequencing (NGS) to determine the epitope of multiple antibodies in parallel in an efficient and comprehensive manner. The method they defined is quite efficient in determining the functional epitope of antibodies but has the disadvantage of being expensive and requires knowledge of NGS and access to the instrumentation. NGS also requires expansive bioinformatics capabilities for the analysis of the data collected.

Here, we have devised a powerful combinatorial mutagenesis type of approach, based on rational grouping of mutated residues to maximize the chances of underscoring combined effects mediated by neighboring residues, displayed on yeast surface, using flow cytometry. One limitation of this approach can be the difficulty in determining whether loss in binding is due to conformational change or due to the disruption of the epitope. We addressed this limitation by conducting parallel experiments using an antibody (5F9-CD3) that does not compete with PF-2610019 for binding to human GUCY2C ECD^40^. Lack of binding of both 5F9 and PF-2610019 to chimeras and patch mutants would indicate a disruptive conformational change in the overall stability of the protein. We found that the antibody PF-2610019 binds to a continuous epitope that forms an alpha helix. A similar method was successfully used for another program in our lab (unpublished), where the epitope was also an alpha helix. We have yet to try this method to find a discontinuous epitope, but this strategy of combining sequence and structural information to design the chimeras and mutants is expected to be capable of also identifying a non-linear epitope.

**Figure 8:**
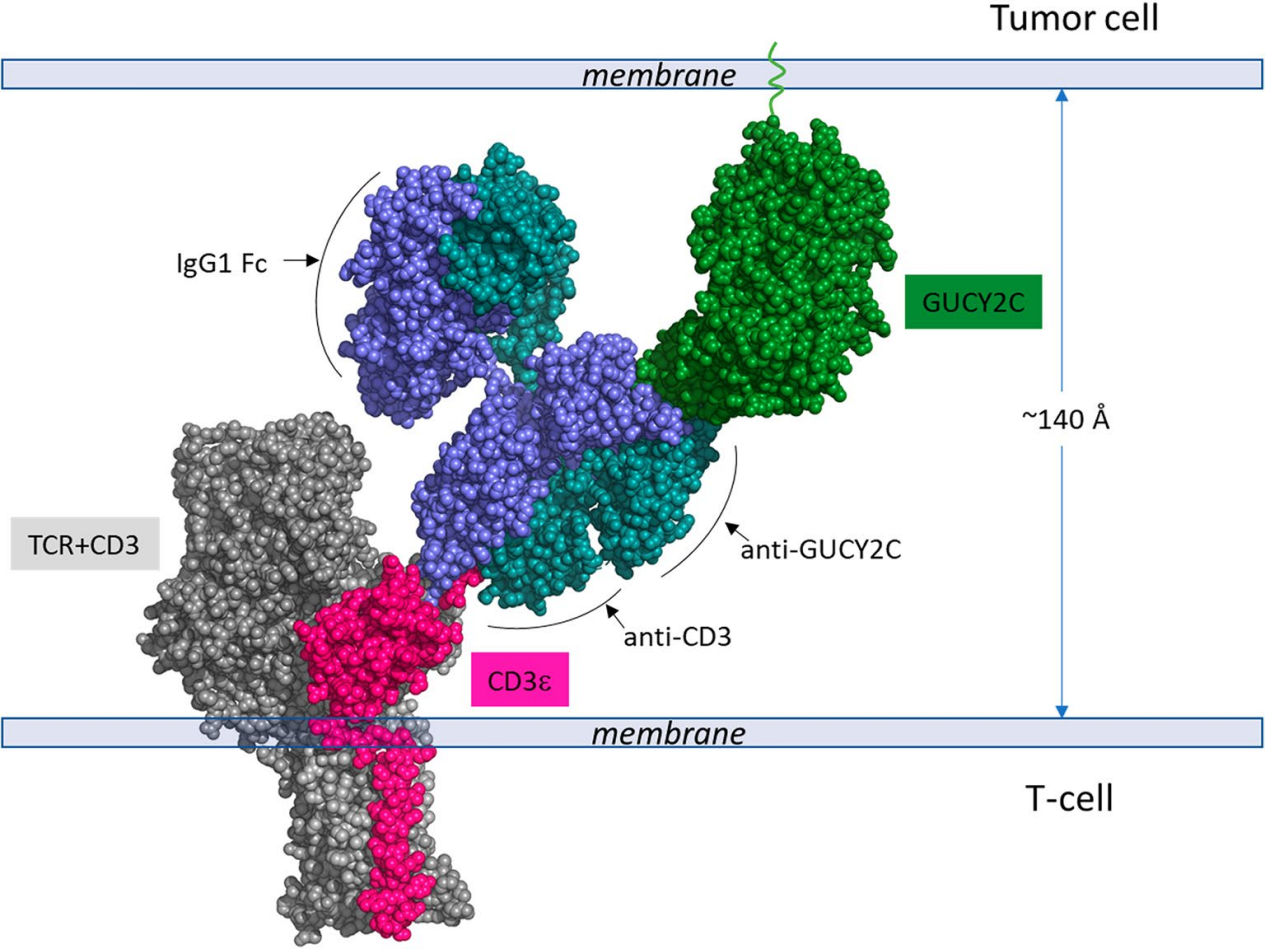
Space-filling model representation of GUCY2C and TCR + CD3 targets on the cell surfaces bound by the CD3xGUCY2C bi-specific antibody. Model depicting the geometry of the extracellular domains of the two targets when bound by the antibody. GUCY2C is colored in green, the bispecific Ab in blue and cyan, and αβTCR + CD3ε in grey and pink. The extrapolated C-terminus of GUCY2C is modelled. The model of the bispecific CD3xGUCY2C is based on the crystal structure of the diabody CD3xGUCY2C fragment that we determined previously^46^. The cryoEM structure of αβTCR + CD3ε (pdb = 7FJE) was used to depict the model of TCR + CD3.

We determined that the anti-GUCY2C arm of PF-2610019 binds exclusively to the N-terminal helix H2 (residues 68–87), targeting the most membrane-distal helical region on GUCY2C (Fig. 7). We and others have shown that optimal cytotoxic T lymphocyte (CTL) killing by CD3 bispecifics can be heavily influenced both by the position and accessibility of the targeted antigen epitope and by a choice of CD3 bispecific modality, including the modular spatial arrangement between the CD3 and the tumor antigen binding arms^18,43–45^*.* To understand the possible impact of the membrane distal location of the GUCY2C epitope on the activity of our CD3xGUCY2C-targeted molecule, which has been constructed in the diabody Fc format as described previously^18^, the data described in the paper allowed us to estimate the apposition of target and effector cell membranes by generating a structure-based model of the CD3xGUCY2C diabody bound to the respective target proteins (Fig. 8). Based on the specific locations of the GUCY2C and CD3ε epitopes and the rigid arrangement of the diabody binding sites, facing opposite from each other, we estimated a distance of about 140 Å between the membranes of the tumor cell and the T-cell (Fig. 8). Such membrane-to-membrane proximity would be consistent with the spacing conferred by other active CD3-bispecifics and by the T cell receptor-MHC complex at the immune synapse^44^. In light of these findings, it is interesting to note that reconfiguration of the same anti-CD3 and anti-GUCY2C binding domains into a more elongated bispecific IgG format showed no CTL killing activity^18^. The two flexible Fab arms of such bispecific IgGs are expected to increase the separation between cells to a suboptimal distance of about 210 Å, underlining the importance of both the antigen epitope location and the chosen modality, the two built-in properties of a CD3 bispecific that can limit its application in bridging T cells to cancer cells.

In summary, the work described here illustrates a powerful alternative approach for identifying the epitopic region of an antibody of interest that is especially useful when the target protein is large, refractory to crystallization, and lacking in available structural information and/or highly similar paralogs. This strategy of substituting regions of the target of interest with sequences from closely-related non-binding orthologs combined with yeast-display and flow cytometry provides a rapid way to localize the antibody binding regions and perhaps allow for finer mapping on a smaller region of the protein than more traditional approaches. In this case, such an approach specifically enabled understanding the mechanism of binding of the CD3xGUCY2C diabody in molecular detail, providing a basis for drug design and optimization.

## Methods

### Yeast surface display

The yeast display vector was generated as previously described by Van Blarcom et al.^23^. A negative control plasmid containing no gene of interest but expressing the V5 tag was also generated. Briefly, proteins of interest were displayed on *S. cerevisiae* strain BJ5465 by transformation of the yeast display vector containing the protein coding sequence using S.C. EasyComp™ Transformation Kit (Invitrogen Cat# K505001) and selected on CM Glucose minus Tryptophan agar plates (Teknova Inc. C3060) for 4 days at 30 °C. Individual colonies were confirmed to contain the vector by PCR using gene specific primers and cultured in SDCAA media (Teknova Inc. 2S0540) overnight at 30 °C, 250 rpm and then shifted to SGCAA (Teknova Inc. 2S0562) media to induce protein expression for 18–24 h at 20 °C, 250 rpm. Cells were harvested, washed with ice-cold phosphate-buffered saline (PBS) supplemented with 0.5% bovine serum albumin (PBSB) and resuspended to a final concentration of 3 × 10^7^ cells/ml.

### Yeast expression constructs

The sequences for GUCY2C ECD’s were derived from UniProtKB-Human GUCY2C ECD-P25092 amino acid (AA) 24–430 (GUC2C_HUMAN), rat GUCY2C ECD-P23897 AA 23–429 (GUC2C_RAT), opposum GUCY2C ECD-F7B751 AA 23–421 (F7B751_MONDO), platypus GUCY2C ECD-F6W2A3 AA 24–432 (F6W2A3_ORNAN), chicken GUCY2C ECD-E1BZE2 AA 25–429 (E1BZE2_CHICK), *Xenopus* (frog) GUCY2C ECD-P79991 AA 17–423 (P79991_XENLA) and zebrafish GUCY2C ECD-A0A0R4IA00 AA 19–411 (A0A0R4IA00_DANRE).

### Flow cytometry

Yeast cells displaying different forms of GUCY2C were incubated with the in-house generated anti-GUCY2C × CD3 bispecific, PF-07062119 at 0.1 μg/ml or 5F9 × CD3 bispecific at 5 μg/ml and anti-V5 antibody (Abcam ab27671-Cambridge, MA) at 1:500 in PBSB for 1 h at room temperature with gentle agitation. The antibody concentration was determined based on titration experiments performed on yeast expressing the human GUCY2C wild-type ECD. The cells were washed with PBSB then incubated with goat anti-human IgG PE at 1:100 dilution (Southern Biotech 2040-09-Birmingham, AL) or goat anti-mouse IgG PE at 1:500 dilution (Southern Biotech 1030-09) in ice-cold PBSB in the dark for 30 min at 4 °C with agitation. Cells were washed with ice-cold PBSB prior to analysis performed on a LSRFortessa with a high-throughput 96-well plate sampler (Becton Dickinson, Franklin Lakes, NJ). Data analysis was performed using FlowJo (Tree Star Inc., Ashland, OR), Excel (Microsoft, Redmond, WA) and GraphPad Prism (San Diego, CA). Median Fluorescence Intensity (MFI) was measured. To normalize binding of anti-GUCY2C antibodies to the expression of the different constructs, the ratio of MFI of the anti-GUCY2C antibodies to the MFI of the anti-V5 antibody was calculated.

### Peptide-based ELISA

Peptides were synthesized and provided as arrays in lyophilized format (New England Peptide, Gardner MA). Arrays were reconstituted with acetonitrile to 100 µg/ml and were then further diluted to 20 µg/ml with sodium carbonate/bicarbonate buffer. Purified antibodies or GUCY2C-CD3 bispecifics were then tested by DELFIA for binding to the different peptides using methods previously reported, with diluted peptides used as the target antigen^18^.

### Human GUCY2C ECD generation

Expi293F™ Cells (ThemoFisher #A14527) maintained in Expi293 expression medium (ThemoFisher #A1435101) shaking at 120RPM at 36 °C and 8% CO_2_ were adjusted to 3e6 cells/ml on day of transfection. Expression plasmid DNA (HuGUCY2C_ECD_TEV_muIgG2amutFc_Flag) and Polyethylenimine Hydrochloride (PEI) (PolySciences #24765) were diluted separately into OptiMEM (ThemoFisher #31985) before combining at a ratio of 1.3:2.6 mg/l and adding to cells. Three hours following transfection valproic acid (Sigma #P4543) was added to a final concentration of 3 mM. Five days following transfection conditioned media was clarified by centrifugation at 1000×*g* for 10 min then filtered by 0.8/0.02 µm depth filtration (Sartopore 2XL). Human GUCY2C ECD was expressed in the presence of a final concentration of 10 µM Kifunensine (Kif) (LC Scientific #KZ300), added at the same time as valproic acid. HuGUCY2C_ECD_TEV_muIgG2amutFc_Flag was purified by batch binding conditioned media to MAb Select Sure (GE Healthcare), washing resin with PBS and eluting with a 20 column volume (CV) gradient from PBS to 20 MM Citric Acid, 150 mM NaCl, pH 2.5. All fractions were neutralized with 10% (final volume) 1 M Tris pH 8. Fractions containing HuGUCY2C_ECD_TEV_muIgG2amutFc_Flag were pooled and concentrated on a 30 K cutoff Vivaspin concentrator. The protein was then further purified by size exclusion chromatography on a Superdex 200 SEC column (GE Healthcare) equilibrated in PBS. The C-terminal Fc and Flag tags were then removed from the protein by digestion with AcTEV Protease (Invitrogen 91636) for 5 h at room temperature. The Fc was removed by binding the protein to MAb Select Sure resin (GE Healthcare) and the TEV was removed by binding to Ni NTA Sepharose FF (GE Healthcare).

### Competition ELISA

A competition assay was performed using a modified version of the DELFIA competition ELISA method. Human GUCY2C ECD protein generated in-house described above was coated on the plate at 1 µg/ml. GUCY2C-1608 (IgG version of PF-07062119) or control antibody at EC_80_ was mixed with serially diluted NSGDCRSSTCEGLDLLRKIS peptide at 10 µg/ml diluted 1:3. Plates were washed 3× with PBST (PBS + 0.05% Tween), and a secondary anti-human IgG europium-conjugated antibody (Perkin Elmer 1244-330) diluted 1:1000 was added for detection. TRF signal was detected on an Envision plate reader with excitation at 320 nm and emission at 615 nm following the manufacturer’s methods. A decrease in TRF signal indicates competitive binding as the Ab GUCY2C-1608 is displaced from binding to human GUCY2C.

### PF-07062119 bispecific and the single chain Fv (aGUCY2C-scFv) Generation

Expi293F™ Cells (ThemoFisher #A14527) maintained in Expi293 expression medium (ThemoFisher #A1435101) shaking at 120RPM at 36 °C and 8% CO_2_ were adjusted to 3e6 cells/ml on day of transfection. Expression plasmid DNA (aGUCY2C-scFv_TEV_Fc) and PEI (PolySciences #24765) were diluted separately into OptiMEM (ThemoFisher #31985) before combining at a ratio of 1.3:2.6 mg/l and adding to cells. Three hours following transfection valproic acid (Sigma #P4543) and Kif (LC Scientific #KZ300) was added to a final concentration of 3 mM and 10 µM, respectively. Five days following transfection conditioned media was clarified by centrifugation at 1000×*g* for 10 min then filtered by 0.8/0.02 μm depth filtration (Sartopore 2XL). aGUCY2C-scFv_TEV_Fc was purified by batch binding media to MAb Select Sure (GE Healthcare), washing resin with PBS and eluting with a 20 CV gradient from PBS to 150 mM Glycine, 40 mM NaCl, pH 3.5. All fractions were neutralized with 10% (final volume) 1 M Tris pH 8. Fractions containing aGUCY2C-scFv_TEV_Fc were then further purified on a Superdex 200 SEC column (GE Healthcare), equilibrated in PBS. The Fc was removed by cleaving the protein with AcTEV Protease (Invitrogen 91636) for 4 h at room temperature followed by binding the protein to MAb Select Sure resin (GE Healthcare) and recovering the unbound fraction. TEV was removed by binding to Ni NTA Sepharose FF (GE Healthcare).

PF-07062119 bispecific was generated as previously described by Root et al.^18^.

### Generation of complex of aGUCY2C-scFv with human GUCY2C ECD

Purified aGUCY2C-scFv and human GUCY2C ECD proteins were mixed at a 1.2:1 molar ratio and allowed to complex at room temperature for 30 min. N-linked glycosylations were removed by incubating with endoglycosidase H for 2 h at 37 °C. The complex was then purified on a Superdex 200 analytical SEC column in TBS. Fractions containing the complex were then concentrated to 11.5 mg/ml with a Vivaspin concentrator.

### Crystallization and structure determination of aGUCY2C-scFv of PF-07062119 complexed with GUCY2C-peptide

For co-crystallization trials, a complex of the anti-GUCY2C single chain Fv (aGUCY2C-scFv) fragment of PF-07062119 and GUCY2C-peptide ^68^NSGDCRSSTCEGLDLLRKIS^87^ (mature sequence numbering) was formed at 1:1.2 molar ratio and was concentrated to 8.8 mg/ml in a protein solution in TBS at pH 7.5. Crystals were obtained by hanging-drop vapor-diffusion method from a condition containing 20% PEG 3350, 200 mM Lithium Sulfate, bis–tris pH 5.5. The rod-like crystals had symmetry consistent with monoclinic space group P2_1_2_1_2_1_ with two copies of complexes in the crystallographic asymmetric unit. A data set to 1.6 Å resolution was collected from a single frozen crystal at IMCA beamline 17-ID at the Argonne National Laboratory (APS). The data were processed and scaled using autoPROC. The structure was solved by molecular replacement with PHASER starting with the model of aGUCY2C-scFv derived from the structure of aGUCY2C-aCD3-diabody that we determined previously^18,46^. The solution was obtained by searching for the two copies of aGUCY2C-scFv. The resulting electron density maps calculated with the two copies of aGUCY2C-scFv as a model unambiguously showed extra electron densities for the two GUCY2C-peptides, each bound to the corresponding copy of aGUCY2C-scFv. The fit of the GUCY2C-peptide into the 2Fo-Fc omit map in which the peptide was not included in phase calculation and into the final 2Fo-Fc map calculated with the complete model shows excellent quality of the electron densities in both maps (Supplementary Fig. S6a,b).

Several iterative rounds of manual adjustment and model rebuilding using COOT and crystallographic refinement using autoBUSTER. This yielded the final model of scFv + GUCY2C-peptide with a crystallographic R_work_ of 19.7% and R_free_ of 21.7%, where R_work_ =||F_obs_| − |F_calc_||/|F_obs_| and R_free_ is equivalent to R_work_, but calculated for a randomly chosen 5% of reflections omitted from the refinement process. Crystal data and refinement statistics are summarized in Supplementary Table S5.

### Crystallization and structure determination of aGUCY2C-scFv complexed with GUCY2C-ECD

For crystallization trials, the purified complex of aGUCY2C-scFv and GUCY2C-ECD (encompassing residues 1–407, mature sequence numbering) was concentrated to 11.3 mg/ml in a protein solution containing TBS at pH 7.5. The conditions that yielded best (3.52 Å) diffracting crystals contained 2 M ammonium H_2_-PO_4_, 100 mM Tris hydrochloride at pH 8.5. The crystals had trigonal symmetry consistent with rhombohedral space group H3, with one copy of complex in the crystallographic asymmetric unit. The diffraction data were collected at IMCA beamline 17-ID at APS and integrated and scaled using autoPROC.

The co-structure was solved by molecular replacement with PHASER starting with the model of aGUCY2C-scFv derived from the structure of the aGUCY2C-scFv + ^68^GUCY2C-peptide^87^ complex. The found solution was fixed and the search was repeated using a model of human GUCY2C-ECD from the AlphaFold protein structure prediction database^47^ in which helices H2 and H3 and the adjacent loop regions were omitted. The initial phasing and refinement were performed with autoBUSTER yielding electron density maps of sufficient quality (Supplementary Fig. S6c) into which the omitted structural regions could be placed unambiguously: helix H2 encompassing ^68^GUCY2C-peptide^87^, the preceding linker between strand S2 and helix H2 (residues 62–67) and helix H3 (residues 99–113). Several iterative rounds of manual adjustment and model rebuilding using COOT and further refinement with autoBUSTER yielded the final model of scFv + GUCY2C with a crystallographic R_work_ of 27.4% and R_free_ of 28.2%. Crystal data and refinement statistics are summarized in Supplementary Table 5.

### Statistics

Data were analyzed using GraphPad Prism 8.1.0 (GraphPad Software, La Jolla, CA, USA). An ANOVA with the Dunnet’s multiple comparisons test were used for multiple experimental groups.

### Supplementary Information

Supplementary Information.

## Data Availability

The sequences for GUCY2C ECD’s were derived from UniProtKB-P25092 (GUC2C_HUMAN), P23897 (GUC2C_RAT), F7B751 (F7B751_MONDO), F6W2A3 (F6W2A3_ORNAN), E1BZE2 (E1BZE2_CHICK), P79991 (P79991_XENLA) and A0A0R4IA00 (A0A0R4IA00_DANRE). The structures of GUCY2C-pep + scFv and GUCY2C-ECD + scFv were deposited to the Protein Data Bank with the following accession codes PDB ID = 8GHO and PDB ID = 8GHP.
