# Supplementary material for: Molecular insights into recognition of GUCY2C by T-cell engaging bispecific antibody anti-GUCY2CxCD3
Source: Sci Rep. 2023 Aug 17;13:13408. doi: 10.1038/s41598-023-40467-0 (PMC10435522; doi:10.1038/s41598-023-40467-0)
Supplement: Supplementary file 1 — Supplementary Information. [file 41598_2023_40467_MOESM1_ESM.pdf]

Molecular insights into recognition of GUCY2C by T-cell engaging bispecific antibody anti-GUCY2CxCD3

Pragya Rampuria[a*#], Lidia Mosyak[a*#], Adam R. Root[b], Kristine Svenson[a], Michael J Agostino[c], Edward R LaVallie[a]

[a]Biomedicine Design, Pfizer Inc., 610 Main St., Cambridge, MA, USA; [b]Generate Biomedicines Inc, Cambridge MA, USA; [c]Pfizer Digital, Pfizer Inc., 1 Burtt Rd, Andover, MA, USA

[#]Pragya Rampuria and Lidia Mosyak contributed equally to the article

*Corresponding authors Pragya Rampuria and Lidia Mosyak. 610 Main St., Cambridge MA, 02139; Email: pragya.rampuria@pfizer.com and lidia.mosyak@pfizer.com

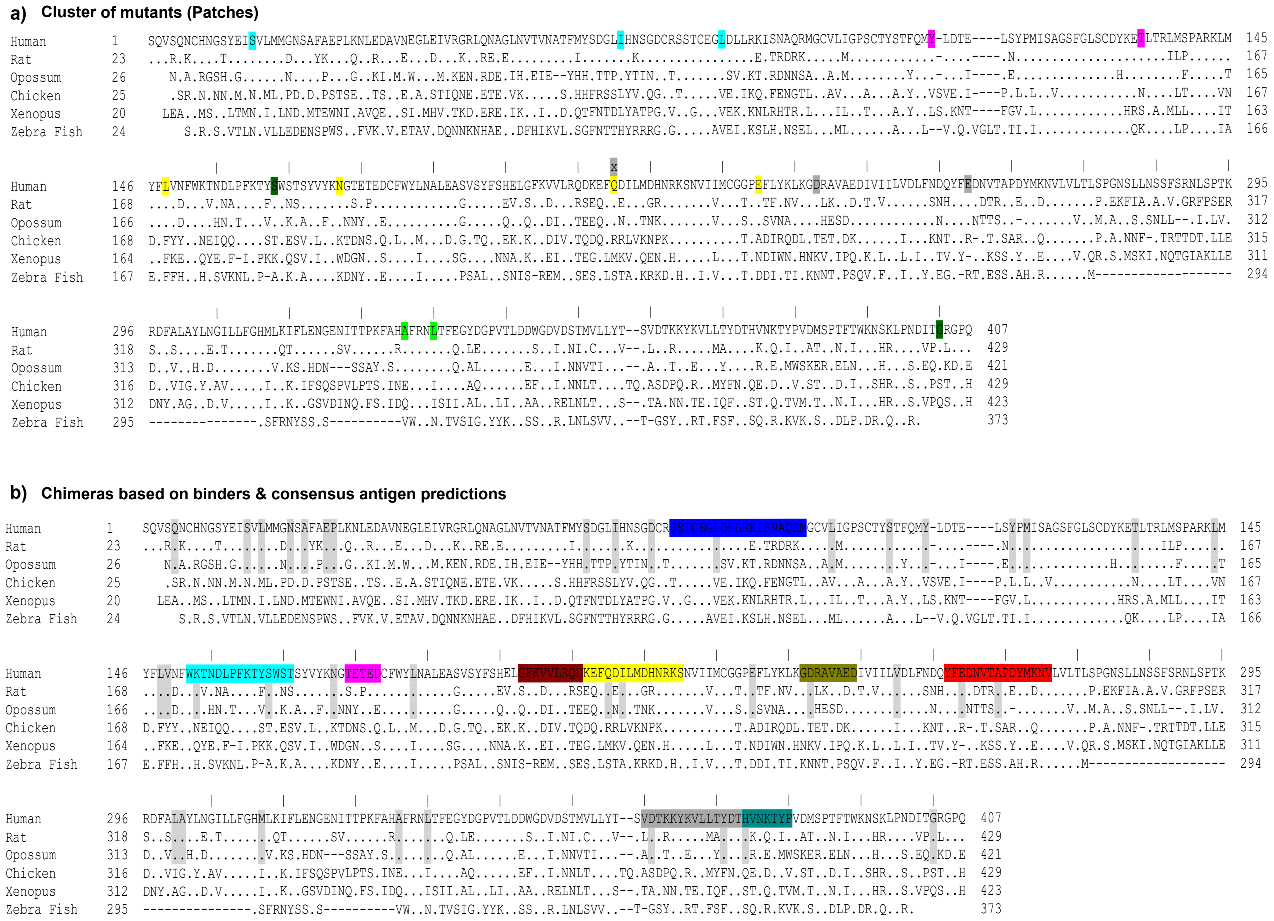

Supplementary Fig. S1: Generation of Patch mutants and chimeras

Multiple sequence alignments of human, rat, opossum, chicken, Xenopus, and zebrafish extracellular (ECD) domains of GUCY2c, created by the NCBI BLASTP algorithm. Residues matching the human sequence are represented by a dot, and differences are shown as letters. Positions in common between "binders" (human, rat, and opossum sequences shown in Fig.1 to bind anti-GUCY2C-CD3 bispecific antibody) and differing in chicken, Xenopus, and zebrafish ('non-binders") are shaded. **a)** Different patch mutants are designated by the residues highlighted in different colors. For instance, all residues highlighted in cyan are part of one patch mutant. **b)** Antigen predictions were performed using three different algorithms at the IEDB Analysis Resource. The different tools were: Bepipred Linear Epitope Prediction, Emini Surface Accessibility Prediction and Kolaskar & Tongaonkar Antigenicity. Of the top-scoring sequences detected by these tools, only regions with overlapping predictions of all three tools were highlighted in the multiple sequence alignment. Each chimera is highlighted in a different color.

a)

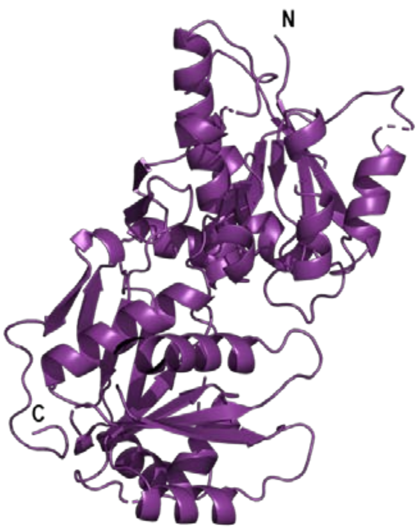

Model based on pdb=1ky0 (Atrial natriuretic peptide clearance receptor)

b)

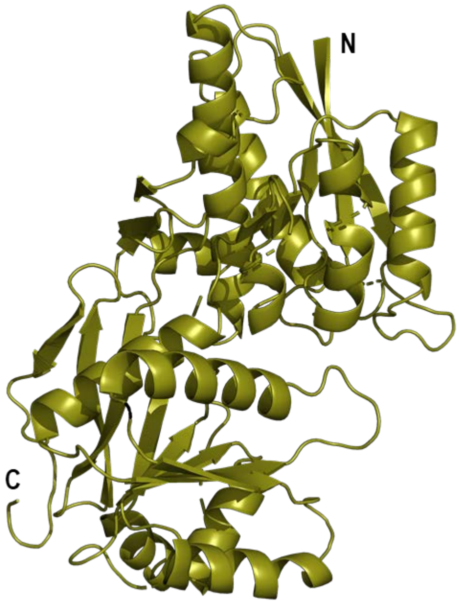

Model based on pdb=1dp4 (Atrial natriuretic peptide receptor A)

Supplementary Fig. S2: Models of GUCY2C-ECD The models were built using FFAS search server http://ffas.godziklab.org/ffas-cgi/cgi/ffas.pl with Templates from the closest homologous structures available in PDB (both) **a)** pdb=1ky0 and **b)** pdb=1dp4 share 17% sequence identity with GUCY2C-ECD). The models were used for searching surface exposed residues to assist GUCY2C-chimera protein designs for Yeast Display.

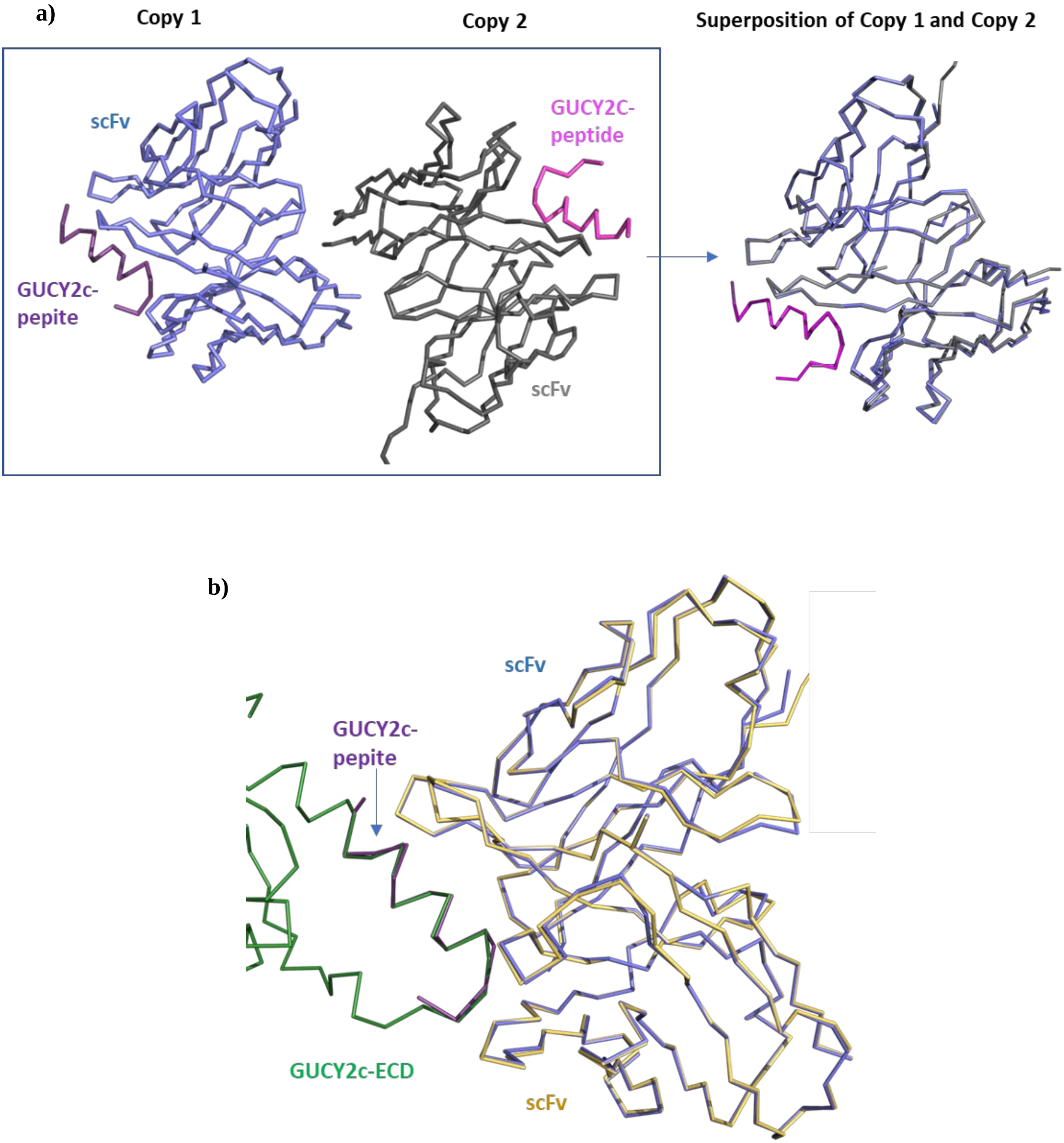

Supplementary Fig. S3. Structural comparison of the GUCY2C-scFv binding interfaces in the two representative crystal structures. a) Ribbon diagram of two independent copies of GUCY2c-peptide+scFv present in the asymmetric unit. The insert on the right is the superposition of the two copies showing a very close match between the two copies. **b)** Superposition of the GUCY2C-peptide+scFv structure (magenta and blue ribbons) and GUCY2C-ECD+scFv structure (green and yellow ribbons) showing remarkable similarity between the two binding interfaces. Same view as in a) for Copy 1.

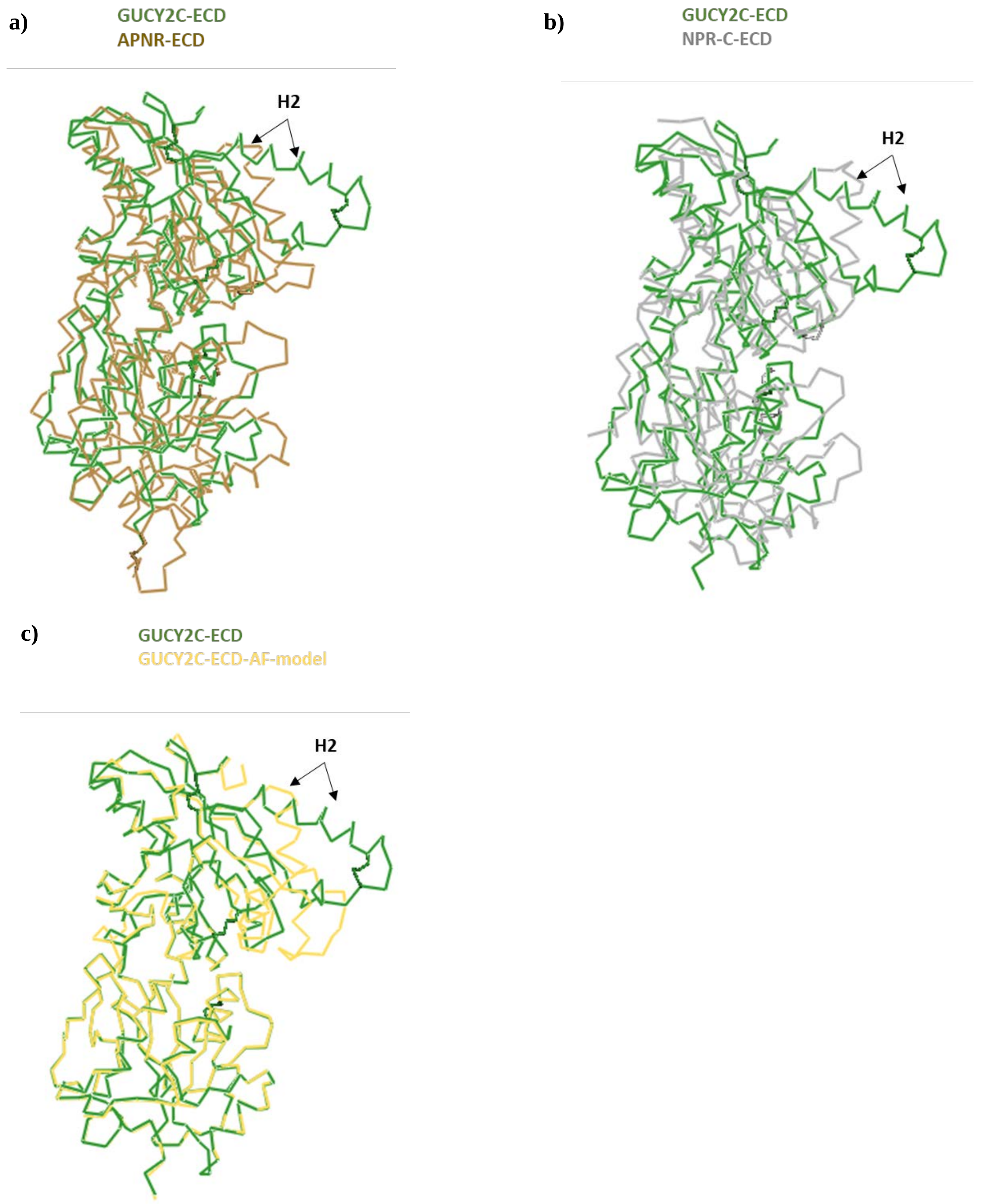

Supplementary Fig. S4. Structural superposition of GUCY2C with ANPR, NPR-C and the AF-model. a) Superposition of GUCY2C-ECD structure (green) and APNR structure (brown). **b)** Superposition of GUCY2C-ECD (green) and NPR-C-ECD (grey) **c)** Superposition of GUCY2C-ECD (green) and AF-model (yellow) showing the best fit compared to that between GUCY2C and ANPR or NPR-C.

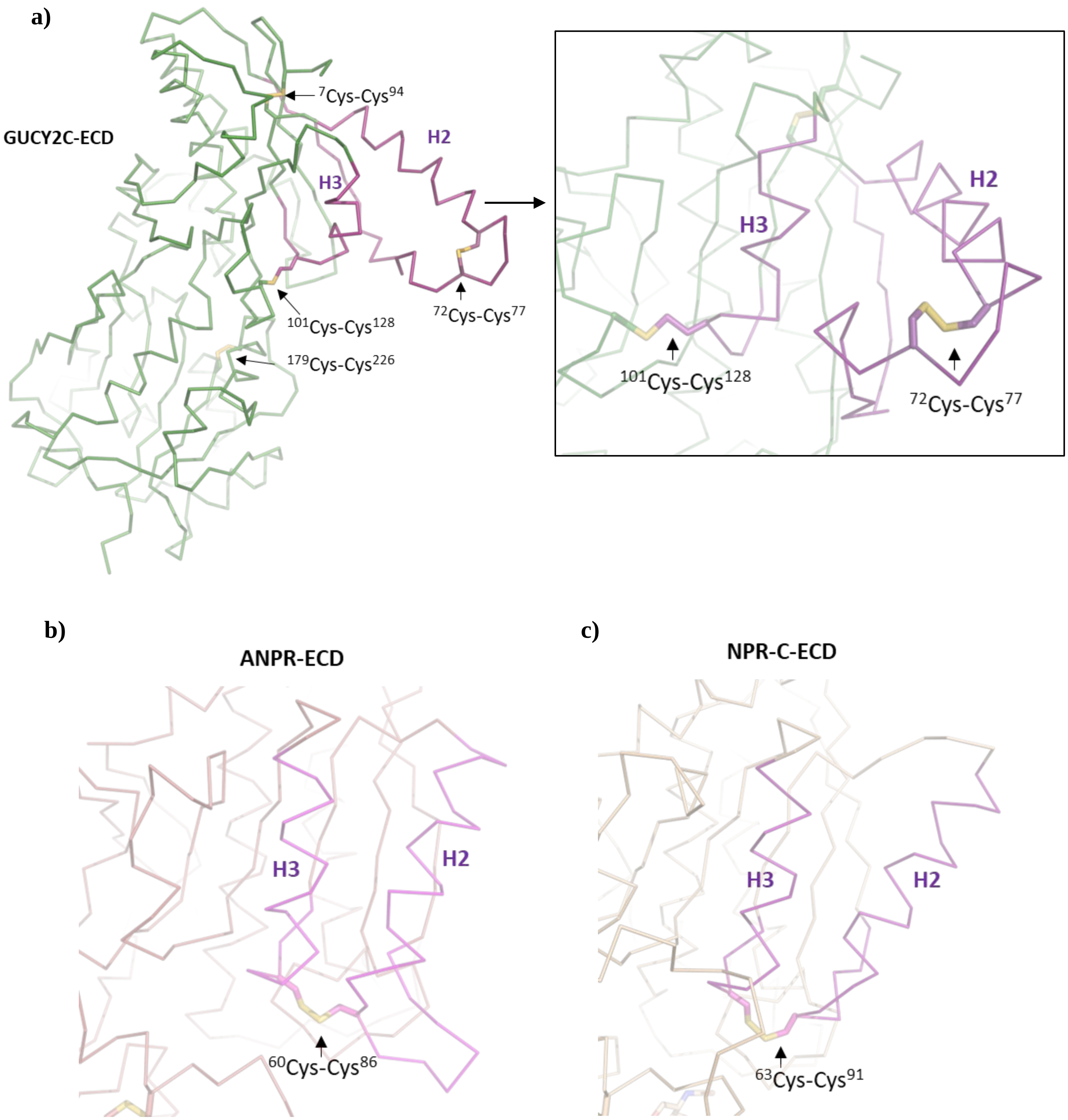

Supplementary Fig. S5. Comparison of disulfide bonds patterns in GUCY2C, ANPR and NPR-C. a) Ribbon diagram of GUCY2C-ECD showing location of four disulfide bridges in the structure. Disulfide bond Cys179-Cys226 located in the C-lobe is the only one that is conserved in ANPR and NPR-C. The remaining three bridges are unique to GUCY2C. The insert on the right is the blow-up view around helices H2 and H3. **b) and c)** Ribbon diagrams of ANPR and NPR-C respectively around helices H2 and H3. Same view as in the insert for GUCY2C. Disulfide bonds are shown as sticks and labeled for each structure.

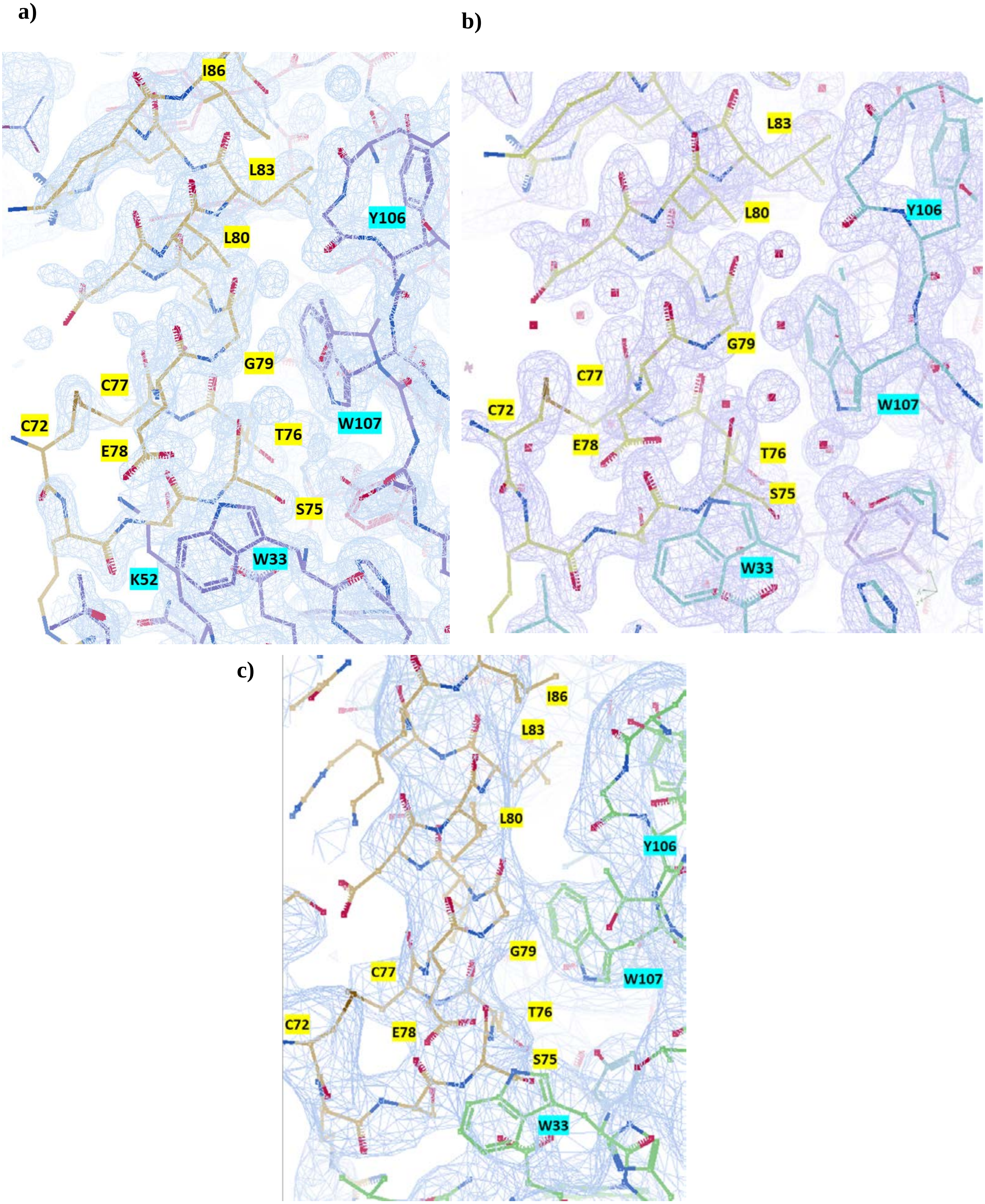

Supplementary Fig. S6 Electron density maps and model fit at the binding interface. a) 1.6 Å resolution 2Fo-Fc omit map, contoured at 1.3σ, in which the GUCY2c-peptide model (yellow sticks) was not included in phase calculation. **b)** The final 1.6 Å resolution 2Fo-Fc map, contoured at 1.3σ. Same view as in a) but calculated with the complete model including water molecules (red dots). **c)** Quality of the 2Fo-Fc map at 3.52 Å resolution, contoured at 1σ and calculated with the complete GUCY2C-ECD+scFv model. Same view as in a) and b). The side chains of interacting residues are labeled in yellow for the peptide and in blue for the heavy chain of scFv in a)-c).

Supplementary table S1: Surface exposed residues determined from models in Supplementary Fig. S2

Amino acid position	Amino acid
21	N
23	A
62	S
66	I
71	D
80	L
109	Y
116	Y
133	T
153	K
163	S
183	L
210	Q
230	E
238	D
259	E
264	A
331	A
335	L
364	D
403	G

Supplementary Table S2: Peptide ELISA

Seq ID	Peptide No.	Peptide Sequence	DELFIA TRF
273	1	MKTLLLDLALWSLLFQPGWL	166.25
274	2	LDLALWSLLFQPGWLSFSSQ	106.5
275	3	WSLLFQPGWLSFSSQVSQNC	97.75
276	4	QPGWLSFSSQVSQNCHNGSY	124.75
277	5	SFSSQVSQNCHNGSYEISVL	108.75
278	6	VSQNCHNGSYEISVLMMGNS	412.5
279	7	HNGSYEISVLMMGNSAFAEP	136
280	8	EISVLMMGNSAFAEPLKNLE	147.5
281	9	MMGNSAFAEPLKNLEDAVNE	145
282	10	AFAEPLKNLEDAVNEGLEIV	105.25
283	11	LKNLEDAVNEGLEIVRGRLQ	101.25
284	12	DAVNEGLEIVRGRLQNAGLN	258.75
285	13	GLEIVRGRLQNAGLNVTVNA	101.5
286	14	RGRLQNAGLNVTVNATFMYS	97
287	15	NAGLNVTVNATFMYSDGLIH	88.75
288	16	VTVNATFMYSDGLIHNSGDC	105.25
289	17	TFMYSDGLIHNSGDCRSSTC	112
290	18	DGLIHNSGDCRSSTCEGLDL	126
291	19	NSGDCRSSTCEGLDLLRKIS	19179.25
292	20	RSSTCEGLDLLRKISNAQRM	3208.75
293	21	EGLDLLRKISNAQRMGCVLI	81
294	22	LRKISNAQRMGCVLIGPSCT	93.5
295	23	NAQRMGCVLIGPSCTYSTFQ	96.25
296	24	GCVLIGPSCTYSTFQMYLDT	118.75
297	25	GPSCTYSTFQMYLDTELSYP	131
298	26	YSTFQMYLDTELSYPMISAG	104.25
299	27	MYLDTELSYPMISAGSFGLS	105
300	28	ELSYPMISAGSFGLSCDYKE	96.75
301	29	MISAGSFGLSCDYKETLTRL	101
302	30	SFGLSCDYKETLTRLMSPAR	125
303	31	CDYKETLTRLMSPARKLMYF	90.25
304	32	TLTRLMSPARKLMYFLVNFW	83.25
305	33	MSPARKLMYFLVNFWKTNDL	87.5
306	34	KLMYFLVNFWKTNDLPFKTY	99
307	35	LVNFWKTNDLPFKTYSWSTS	108
308	36	KTNDLPFKTYSWSTSYVYKN	111.5
309	37	PFKTYSWSTSYVYKNGTETE	106.75
310	38	SWSTSYVYKNGTETEDCFWY	109.5
311	39	YVYKNGTETEDCFWYLNALE	86.5
312	40	GTETEDCFWYLNALEASVSY	85.25
313	41	DCFWYLNALEASVSYFSHEL	161
314	42	LNALEASVSYFSHELGFKVV	96.5
315	43	ASVSYFSHELGFKVVLRQDK	104
316	44	FSHELGFKVVLRQDKEFQDI	90.5
317	45	GFKVVLRQDKEFQDILMDHN	107.5

Supplementary Table S2 Continued: Peptide ELISA

Seq ID	Peptide No.	Peptide Sequence	DELFIA TRF
318	46	LRQDKEFQDILMDHNRKSNV	105.75
319	47	EFQDILMDHNRKSNVIIMCG	103.75
320	48	LMDHNRKSNVIIMCGGPEFL	148
321	49	RKSNVIIMCGGPEFLYKLKG	129.25
322	50	IIMCGGPEFLYKLKGDRAVA	161
323	51	GPEFLYKLKGDRAVAEDIVI	96.75
324	52	YKLKGDRAVAEDIVIILVDL	103.75
325	53	DRAVAEDIVIILVDLFNDQY	87.75
326	54	EDIVIILVDLFNDQYLEDNV	112.25
327	55	ILVDLFNDQYLEDNVTAPDY	100.25
328	56	FNDQYLEDNVTAPDYMKNVL	81
329	57	LEDNVTAPDYMKNVLVLTLS	99.75
330	58	TAPDYMKNVLVLTLSPGNSL	98.5
331	59	MKNVLVLTLSPGNSLLNSSF	135.25
332	60	MKNVLVLTLSPGNSLLNSSF	150.25
333	61	VLTLSPGNSLLNSSFSRNLS	125.5
334	62	PGNSLLNSSFSRNLSPTKRD	91.25
335	63	LNSSFSRNLSPTKRDFALAY	109.5
336	64	SRNLSPTKRDFALAYLNGIL	116
337	65	PTKRDFALAYLNGILLFGHM	103
338	66	FALAYLNGILLFGHMLKIFL	114.25
339	67	LNGILLFGHMLKIFLENGEN	93.25
340	68	LFGHMLKIFLENGENITTPK	73.5
341	69	LKIFLENGENITTPKFAHAF	100.5
342	70	ENGENITTPKFAHAFRNLTF	91.75
343	71	ITTPKFAHAFRNLTFEGYDG	105
344	72	FAHAFRNLTFEGYDGPVTLD	116
345	73	RNLTFEGYDGPVTLDDWGDV	116.25
346	74	EGYDGPVTLDDWGDVDSTMV	100
347	75	PVTLDDWGDVDSTMVLLYTS	88.25
348	76	DWGDVDSTMVLLYTSVDTKK	102
349	77	DSTMVLLYTSVDTKKYKVLL	98.25
350	78	LLYTSVDTKKYKVLLTYDTH	97.75
351	79	VDTKKYKVLLTYDTHVNKTY	130
352	80	YKVLLTYDTHVNKTYPVDMS	1468.75
353	81	TYDTHVNKTYPVDMSPTFTW	109
354	82	VNKTYPVDMSPTFTWKNSKL	99.25
355	83	PVDMSPTFTWKNSKLPNDIT	85.75
356	84	PTFTWKNSKLPNDITGRGPQ	133
357	85	KNSKLPNDITGRGPQILMIA	163.25
358	86	PNDITGRGPQILMIAVFTLT	110.5
359	87	LRKISNAQRMGCVLIGPSCT	102
360	88	NAQRMGCVLIGPSCTYSTFQ	120.25
361	89	GCVLIGPSCTYSTFQMYLDT	115
362		NSGDCRSSTCEGLDLLRKISNAQRM	
363		RSSTCEGLDLLRKIS	

Supplementary Table S3: Amino acids that define Paratope (chains H+L) and Epitope (chain G)

Residues having atoms within 3.80 Angstrom				
GUCY2C chain	GUCY2C Residue	Antibody Chain	Antibody Residue	Electrostatic interactions
G	R73	H	N59	
G	S74	H	W33	
G	S74	H	E50	
G	S74	H	N59	
G	S75	H	W33	
G	S75	H	E50	H-bond
G	S75	L	Y99	H-bond
G	T76	L	T95	
G	T76	L	R96	
G	T76	L	A98	H-bond
G	E78	H	W33	H-bond
G	E78	H	K52	H-bond
G	G79	H	W107	
G	L80	H	W107	
G	L80	L	Y31	
G	L80	L	L36	
G	L80	L	R96	
G	L83	H	E104	
G	L83	H	G105	
G	L83	L	L36	
G	R84	L	Y31	
G	R84	L	Y32	
G	I86	H	E104	
G	I86	H	G105	

Supplementary Table S4: Contribution of [68]Gucy2c-peptide[87] to the interface

Type	Chain GUCY2c	Position	%BSA	Electrostatic Interactions
ARG	C	73	50.28431	H
SER	C	74	56.99118	
SER	C	75	93.22802	H
THR	C	76	85.61988	H
GLU	C	78	46.89699	H
GLY	C	79	86.96493	W
LEU	C	80	78.57378	
LEU	C	82	28.2139	
LEU	C	83	88.59774	
ARG	C	84	40.15275	
ILE	C	86	23.20466	

The amino acids contributing > 75% of their surface areas to the binding interface are highlighted in red and orange.

Supplementary Table S5:
Crystallographic data collection and refinement statistics

	**GUCY2C-peptide + aGUCY2C-scFv**	**GUCY2C-ECD + aGUCY2C-scFv**
Data collection statistics		
Space group	$P2_12_12_1$	H3
Unit cell dimensions<br>a, b, c (Å)<br>α, β, γ (°)	70.7, 80.6, 90.7<br>90, 90, 90	199.6, 199.6, 123.3<br>90, 90, 120
Number of independent copies in ASU	2	1
Wavelength (Å)	1.0	1.0
Resolution (Å)	60.2 – 1.6 (1.77 – 1.6)	99.8 – 3.52 (3.77 – 3.52)
Number of unique reflections	49,431 (2,472)	17,636 (881)
⟨I/σ⟩	12.3 (1.6)	8.3 (0.94)
Completeness (%)	92.9 (61.7)	91.8 (50.1)
Redundancy	6.6 (6.2)	5.2 (5.4)
R_{merge}[a]	0.085 (0.9)	0.12 (0.9)
Refinement statistics		
Resolution (Å)	31.9 – 1.6 (1.64 – 1.6)	34.2 – 3.52 (3.74 – 3.52)
R_{cryst}/R_{free} (%)[b]	19.7 /21.7 (23.5/24.5)	27.4/28.2 (22.1/22.6)
Number of reflections<br>Working set<br>Test set	49,431<br>2,528	17,612<br>655
Completeness (%)	71.4(2.35)	77.8(17.8)
rmsd from ideal values<br>Bond lengths (Å)<br>Bond angles (°)	0.008<br>1.09	0.008<br>1.1
Number of atoms<br>Protein<br>Solvent<br>Heterogen atoms	3,923<br>537<br>0	5,060<br>0<br>42
Mean B values (Å^2)	32.7	105.4
Ramachandran plot[c]<br>Favoured regions (%)<br>Outliers (%)	95.35<br>1.41	89.45<br>2.36
All-atom clashscore	4	8

Numbers in parentheses are for the highest-resolution shell.
[a] Rmerge=Σ|I−⟨I⟩|/ΣI, where I is the intensity of the measured reflection, and ⟨I⟩ is the mean intensity of all measurements of this reflection.
[b] Rcryst=Σ‖F_{obs}|−|F_{calc}‖/Σ|F_{obs}|, where Fobs and F_{calc} are the observed and calculated structure factors, respectively. R_{free} is calculated for 5% of reflections randomly chosen prior to refinement.
[c] The Ramachandran plot was calculated with Coot.
